# Characterization of the gut microbiota and fecal and blood metabolomes under various factors in urban children from Northwest China

**DOI:** 10.3389/fcimb.2024.1374544

**Published:** 2024-03-22

**Authors:** Yan Yang, Juanjuan Chen, Huiyu Gao, Minglu Cui, Mingyu Zhu, Xuesong Xiang, Qi Wang

**Affiliations:** ^1^ Department of Endocrinology and Metabolism, Lanzhou University Second Hospital, Lanzhou, China; ^2^ Cuiying Biomedical Research Center, Lanzhou University Second Hospital, Lanzhou, China; ^3^ National Institute of Nutrition and Health, Chinese Center for Disease Control and Prevention, Beijing, China; ^4^ The Second School of Clinical Medicine, Lanzhou University, Lanzhou, Gansu, China

**Keywords:** children in northwest China, gut microbiota, fecal and plasma metabolome, age, body mass index, regular physical exercise, delivery mode

## Abstract

**Introduction:**

Children have regional dynamics in the gut microbiota development trajectory. Hitherto, the features and influencing factors of the gut microbiota and fecal and plasma metabolites in children from Northwest China remain unclear.

**Methods:**

Shotgun metagenomic sequencing and untargeted metabolomics were performed on 100 healthy volunteers aged 2-12 years.

**Results:**

Age, body mass index (BMI), regular physical exercise (RPE), and delivery mode (DM) significantly affect gut microbiota and metabolites. *Lactobacillus*, *Butyricimonas*, *Prevotella*, *Alistipes*, and predicted pathway propanoate production were significantly increased with age while *Bifidobacterium breve, B. animalis, B. pseudocatenulatum*, *Streptococcus infantis*, and carbohydrate degradation were decreased. Fecal metabolome revealed that the metabolism of caffeine, amino acids, and lipid significantly increased with age while galactose metabolism decreased. Noticeably, BMI was positively associated with pathogens including *Erysipelatoclostridium ramosum*, *Parabacteroides distasonis*, *Ruminococcus gnavus*, and amino acid metabolism but negatively associated with beneficial *Akkermansia muciniphila*, *Alistipes finegoldii*, *Eubacterium ramulus*, and caffeine metabolism. RPE has increased probiotic *Faecalibacterium prausnitzii* and *Anaerostipes hadrus*, acetate and lactate production, and major nutrient metabolism in gut and plasma, but decreased pathobiont *Bilophila wadsworthia*, taurine degradation, and pentose phosphate pathway. Interestingly, DM affects the gut microbiota and metabolites throughout the whole childhood. *Bifidobacterium animalis*, *Lactobacillus mucosae*, *L. ruminis*, primary bile acid, and neomycin biosynthesis were enriched in eutocia, while anti-inflammatory *Anaerofustis stercorihominis*, *Agathobaculum butyriciproducens*, *Collinsella intestinalis*, and pathogenic *Streptococcus salivarius*, *Catabacter hongkongensis*, and amino acid metabolism were enriched in Cesarean section children.

**Discussion:**

Our results provided theoretical and data foundation for the gut microbiota and metabolites in preadolescent children’s growth and development in Northwest China.

## 1 Introduction

The gut microbiome is constantly changing, from infancy to childhood to adolescence (Wehrle et al., 2020). Several variables could affect its development and colonization (Yang et al., 2021). Delivery mode (DM) and feeding patterns are the initial influencing factors of the gut microbiome (Wernroth et al., 2022). Neonatal microbial colonization and maturation of the immune system are thought to occur in parallel to influence intestinal physiology and regulation against infections in early life (Sanidad and Zeng, 2020) and delayed gut microbiota maturation was related to pediatric allergic disease (Hoskinson et al., 2023). Then, the gut was dominated by *Bacteroidetes* but a reduction in Proteobacteria species along with the gradual increase in the gut microbial phylogenetic diversity during the first 3 years of life (Niu et al., 2020). The gut microbiota largely reaches a relatively stable adult-like composition by 3–5 years of age (Guo et al., 2020) and was dominated by *Firmicutes*, *Bacteroidetes*, *Prevotella*, *Faecalibacterium*, *Bifidobacterium*, and *Akkermansia muciniphila* (Guo et al., 2020) (Deering et al., 2019). Children suffering from diseases, including new-onset type 1 diabetes (Yuan et al., 2022), systemic lupus erythematosus (Wen et al., 2021b), Henoch-Schönlein Purpur (HSP) (Wen et al., 2021a), diarrhea (Gallardo et al., 2020), and autism spectrum disorder (Zuffa et al., 2023), were accompanied with dysbiosis and metabolic alterations of the gut microbiota. Compared with adults, an overrepresentation of the glycan degradation and vitamin B2/6/9 biosynthesis in the gut microbiota were observed in children between 9 and 12 years (Radjabzadeh et al., 2020). These lines of evidence reminded us of the importance of studying the gut microbiota and metabolites in children of all ages.

Appropriate physical activity can ensure the presence of a functional physiological microbiota to maintain human health (Wegierska et al., 2022) caused by a sedentary lifestyle, among others. For children, exercise can not only increase food intake but also enhance physical fitness. Childhood obesity has reached epidemic proportions worldwide (Pihl et al., 2016), and exercise training could modulate the gut microbiota profile and impairs inflammatory signaling pathways in obese children (Quiroga et al., 2020). However, the relationship between the gut microbiota and changes in body mass index (BMI) or pediatric overweight in Chinese children, especially from Northwest China, remains unclear.

Geographical factors showed the strongest associations with gut microbiome for regional dietary and living habits (He et al., 2018). The preadolescents from non-Western locations had higher α-diversity and SCFA concentrations (Deering et al., 2019). In children from South Africa, *Firmicutes* and *Bacteroidetes* dominated after infancy and *Prevotella* was the most common genus during the first 5 years of life (Nel et al., 2021). In a Chinese study, children born and fed in Beijing had a higher abundance of *Enterococcaceae* and *Lachnospiraceae*, while children from Shenzhen had a higher abundance of *Fusobacteriaceae* (Niu et al., 2020) in the first 3 years. Another study based on four regions of China revealed that geography and age affect the gut microbiota, and *Bifidobacterium* was enriched in children (Yang et al., 2020).

Taken together, the current studies on gut microbiota and metabolites have obtained some achievements based on 16S rRNA gene sequencing in diseased children. However, fecal metabolome, a functional readout of the gut microbiome (Zierer et al., 2018), and the blood metabolites, which can predict and provide therapeutic targets for diseases (Chen et al., 2023), were lacking in healthy children. China has given birth to rich and unique ecosystems, species, and genetic diversity with its vast territory, complex landscapes, diverse climates, and multi-ethnic inhabitants. The gut microbiota of children from southeast coastal and first-tier cities in China has been explored (Niu et al., 2020; Yang et al., 2020). However, a gap exists in the gut microbiota and microbial and blood metabolites in healthy children from Northwest China. In this study, we aimed to elucidate the use of shotgun metagenomic sequencing and untargeted metabolomics in healthy children aged 2–12 years from Northwest China. Our study would be of particular relevance to the gut microbiota and metabolites in children’s development and health, providing theoretical basis and important references for the healthcare of Chinese children from Northwest regions.

## 2 Materials and methods

### 2.1 Study design and ethics statement

This study was designed to investigate the composition and function of the gut microbiota and fecal and blood metabolites in healthy children from Northwest China using shotgun metagenomic sequencing and untargeted metabolomics.

The study was conducted in accordance with the Declaration of Helsinki and approved by the Ethics Committee of Lanzhou University Second Hospital (protocol code 2022A-221, date of approval: 2 March 2022).

### 2.2 Subject recruitment and sample collection

The inclusion criteria of the subjects were as follows: (1) they should be outwardly healthy and have no known diseases such as chronic infection; (2) they should have a theoretical age between 3 and 12 years; (3) they should have had no antibiotics or other medications that may affect the gut microbiota for nearly a month; (4) they should have had no intake of fermented foods such as pickles and yogurt in nearly 1 month; (5) they should have been weaned; the diet is mainly based on rice, cooked wheaten food, meat, and vegetables, and a daily intake of less than 300 mL of milk; and (6) they should have complete information on samples and phenotypes.

The basic information of the standard-compliant children was collected using a designed questionnaire including age, sex, birth weight and height, DM, current body weight and height, physical exercise, and dietary intake (**Supplementary Table S1A**). Finally, 100 healthy Chinese children with complete phenotypic information were recruited from Xigu District (Lanzhou, Gansu) in this study.

Fresh fecal samples (5–10 g) were collected from participants and stored at −80°C for shotgun metagenomics and metabolomics. Overnight fasting venous blood was collected from each participant to centrifuge at 3,000 rcf (×*g*) for 10 min to obtain plasma and stored at −80°C for metabolomics.

### 2.3 Shotgun metagenomic sequencing and data analysis

#### 2.3.1 DNA extraction, quantification, sequencing, and filtering

All fecal samples were transferred by dry ice in the laboratory to extract DNA. Total DNA from each sample was extracted in accordance with the protocol as described previously (Fang et al., 2018). The quality and quantity of the DNA were measured using NanoDrop Spectrophotometer ND-1000 (Thermo Fisher Scientific Inc.). Metagenome library was constructed using the TruSeq DNA PCR-Free Library Preparation Kit (Illumina), and the quantity of each library was evaluated using a Qubit 2.0 fluorimeter (Invitrogen). Sequencing of metagenome libraries was conducted at BGI-Shenzhen (Shenzhen, China) using BGI-Seq500 (150-bp paired-end sequencing of ~ 350-bp inserts) (Fang et al., 2018).

The raw reads that had 50% low-quality bases (quality ≤ 20) or more than five ambiguous bases were excluded using FASTP. The remaining reads were mapped to human genome (hg19) by bowtie2 (-m 100- × 600 -v 7 -p 6 -l 30 -r 1 -M 4 -c 0.95), and the matching reads were removed. The high-quality reads (clean reads) were used for taxonomic and functional analysis.

#### 2.3.2 Taxonomic profiling

Metagenomic Phylogenetic Analysis (MetaPhlAn, version 3.0, - input_type fastq - ignore_viruses - nproc 6) as described in the reference was used to generate phyla, genera, and species profiles from the clean reads as previously reported (Beghini et al., 2021). MetaPhlAn is a computational tool for profiling the composition of microbial communities from metagenomic shotgun sequencing data. MetaPhlAn relies on unique clade-specific marker genes identified from ~17,000 reference genomes (~13,500 bacterial and archaeal, ~3,500 viral, and ~110 eukaryotic), allowing up to 25,000 reads-per-second (on one CPU) analysis speed; unambiguous taxonomic assignments as the MetaPhlAn markers are clade-specific; accurate estimation of organismal relative abundance; species-level resolution for bacteria, archaea, eukaryotes, and viruses; and extensive validation of the profiling accuracy on several synthetic datasets and on thousands of real metagenomes.

#### 2.3.3 Functional profiling

The HMP Unified Metabolic Analysis Network (HUMAnN, version 3.0, -i input_clean_data -o output –threads 10 –memory-use maximum –remove-temp-output) is used for predicted functional metagenome analysis as previously reported (Beghini et al., 2021). HUMAnN is a method for efficiently and accurately profiling the abundance of microbial metabolic pathways and other molecular functions from metagenomic sequencing data.

#### 2.3.4 Diversity analysis

α-Diversity [R 4.0.3 vegan: diversity (data, index = ‘ richness/Shannon/Simpson/InSimpson’)] was calculated using the richness, Shannon index, Simpson’s index, and Inverse Simpson’s index, depending on the taxonomic profiles. β-Diversity [(R 4.0.3 ape: pcoa (‘bray_curtis distance’, correction=“none”, rn=NULL)) between-sample diversity, R 4.0.3 vegan: diversity (data, index = ‘bray_curtis distance’)] was calculated using the Bray–Curtis distance depending on the taxonomic profiles. Permutational Multivariate Analysis of Variance [PERMANOVA; code: R 4.0.3: adonis (dist~phe, permutations = 1,000)] was performed based on the gut microbial species/genus abundance profile to study the effect on the gut microbiome.

#### 2.3.5 Partial correlation analysis

Partial correlation analysis involves studying the linear relationship between two variables after excluding the effect of one or more independent factors and was used for correlation analysis between age and BMI and species, genera, phyla, and Kyoto Encyclopedia of Genes and Genomes (KEGG) pathways.

#### 2.3.6 Differential analysis

The significant species, genera, phyla, and KEGG pathways between children with eutocia and Cesarean section (C-section), and children with and without regular physical exercise (RPE) were analyzed.

### 2.4 Untargeted metabolomics

#### 2.4.1 Sample preparation and extraction

Fecal and plasma samples were freeze-dried using a vacuum freeze-dryer (Scientz-100F) and then were crushed in a mixer mill (MM 400, Retsch) with zirconia bead for 1.5 min at 30 Hz. The lyophilized powder (50 mg) was mixed in 1.2 mL of 70% methanol solution and vortexed six times for 30 s every 30 min. After centrifugation at 12,000 rcf (×*g*) for 3 min, the extracts were filtered (SCAA-104, 0.22 μm pore size; ANPEL, Shanghai, China, http://www.anpel.com.cn/) and then subjected to ultraperformance liquid chromatography-tandem mass spectrometry (UPLC-MS/MS).

#### 2.4.2 UPLC conditions

The sample extracts were analyzed using a UPLC-ESI-MS/MS system (UPLC, SHIMADZU Nexera X2; MS, Applied Biosystems 6500 Q TRAP). The analytical conditions were as follows. UPLC: column, Agilent SB-C18 (1.8 µm, 2.1 mm × 100 mm). The mobile phase consisted of solvent A (pure water with 0.1% formic acid) and solvent B (acetonitrile with 0.1% formic acid). Sample measurements followed a gradient program starting with 95% A and 5% B. Within 9 min, a linear gradient transition of 5% A and 95% B was programmed, and was maintained for 1 min. Subsequently, the composition was adjusted to 95% A and 5.0% B within 1.1 min and maintained for 2.9 min. The flow velocity was set to 0.35 mL per minute; The column oven temperature was set to 40°C, and the injection volume was 2 μL. The effluent was alternately connected to an electrospray ionization triple quadrupole linear ion trap (QTRAP)-MS system.

#### 2.4.3 ESI-Q TRAP-MS/MS

The electrospray ionization (ESI) source operation parameters were as follows: source temperature, 500°C; ion spray voltage (IS), 5,500 V (positive ion mode)/−4,500 V (negative ion mode); ion source gas I (GSI), gas II (GSII), and curtain gas (CUR) set at 50, 60, and 25 psi, respectively; and high collision-activated dissociation (CAD). Instrument tuning and mass calibration were performed using 10 and 100 μmol/L polypropylene glycol solutions in the QQQ and LIT modes, respectively. QQQ scans were acquired in the MRM experiments using a collision gas (nitrogen) set in the medium. The declustering potential (DP) and collision energy (CE) for individual MRM transitions were determined by DP and CE optimization. A specific set of MRM transitions was monitored for each period according to the metabolites eluted within this period.

#### 2.4.4 Principal coordinate analysis of metabolites

Principal coordinate analysis (PCoA) was performed using the ape comp statistical function in R (www.r-project.org) based on Bray–Curtis distances of the fecal and plasma metabolites.

#### 2.4.5 Differential metabolites selected

Significantly differentially regulated metabolites between obese and control mice were determined using the Wilcoxon rank-sum test (*P* < 0.05).

#### 2.4.6 KEGG annotation and enrichment analysis

Identified metabolites were annotated using the KEGG compound database (http://www.kegg.jp/kegg/compound/) and annotated metabolites were mapped to the KEGG Pathway database (http://www.kegg.jp/kegg/pathway.html).

### 2.5 Statistical methods

PERMANOVA was used to study the effect of various phenotypes on the gut microbiota and fecal and plasma metabolites. Spearman’s rank correlation was used to analyze the relationship between age and multiomics. Partial correlation analysis was used for correlation analysis between BMI and multiomics to adjust the effect of age and sex. Wilcoxon rank-sum test was used to compare the difference of the species, genera, phyla, KEGG pathways, and fecal and plasma metabolites between eutocia and C-section, as well as with and without RPE, before which partial correlation analysis was used to adjust the influence of age, sex, and BMI. Spearman’s rank correlation analysis was used to analyze the associations between multiomics under different influencing factors.

All statistical analyses were based on R packages (Version 4.2.1). PERMANOVA: vegan, adonis(t(otu1) ~ phe (,^1^), data = phe, permutations = 999, na.rm = T). Wilcoxon rank-sum test: wilcox.test(as.numeric(pr[i, f1]), as.numeric(pr[i, f2])). Heatmap: pheatmap(cmt,scale = “none”,cluster_row = T, cluster_col = T,display_numbers = pmt). Partial correlation analysis: ppcor, pcor.test(y.data (,^1^),y.data (,^2^),y.data[,c(3:5)],method = “spearman”). *P* < 0.05 was considered significant difference.

## 3 Results

### 3.1 Overview of the gut microbiota and fecal and plasma metabolites

A total of 100 children aged between 2 and 12 years old with an average age of 5.59 ± 2.26 years were recruited in this study. The subjects were sex-matched, with 48 girls and 52 boys included in the study.

**Figure 1:**
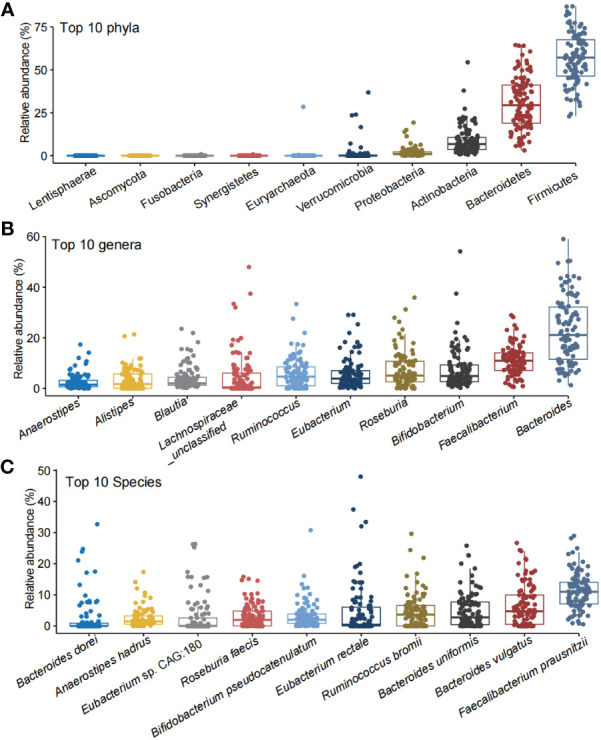
The top 10 abundant phyla, genera, and species in the healthy children. The top 10 phyla **(A)**, genera **(B)**, and species **(C)** in the gut of the the healthy children.

MetaPhlAn 3.0 has revealed 11 phyla, 178 genus, and 501 species in the subjects. The α-diversity for species and genus was evaluated by Shannon, Simpson, and Inverse Simpson indexes. The Shannon, Simpson, and Inverse Simpson indexes for genus and species were 2.35 ± 0.27, 0.84 ± 0.06, and 6.88 ± 2.30, and 2.92 ± 0.30, 0.90 ± 0.05, and 11.33 ± 3.79, respectively (**Supplementary Table S1B**). *Firmicutes*, *Bacteroidetes*, Actinobacteria, Proteobacteria, and Verrucomicrobia were the top five abundant phyla (**Figure 1A**). *Anaerostipes*, *Alistipes*, *Blautia*, *Ruminococcus*, *Eubacterium*, *Roseburia*, *Bifidobacterium*, *Faecalibacterium*, *Bacteroides*, and *Lachnospiraceae_unclassified* were the top 10 abundant genera (**Figure 1B**). *Bacteroides dorei*, *Anaerostipes hadrus*, *Eubacterium* sp. CAG:180, *Roseburia faecis*, *Bifidobacterium pseudocatenulatum*, *Eubacterium rectale*, *Ruminococcus bromii*, *Bacteroides uniformis*, *Bacteroides vulgatus*, and *Faecalibacterium prausnitzi* were the top 10 abundant species (**Figure 1C**).

HUMAnN 3.0 was used to profile the abundance of microbial metabolic pathways from metagenomic sequencing data. A total of 486 predicted functional pathways were obtained. The top 10 abundant predicted pathways were starch degradation V, adenosine ribonucleotides *de novo* biosynthesis, pyruvate fermentation to isobutanol (engineered), dTDP-β L-rhamnose biosynthesis, L-isoleucine biosynthesis I (from threonine), L-valine biosynthesis, UMP biosynthesis I, glycolysis IV, UMP biosynthesis I, and adenine and adenosine salvage III (**Supplementary Figure S1**). Interestingly, *F. prausnitzi* has participated in all the top 10 abundant predicted functional pathways, suggesting the importance of this bacterium in children from Northwest China.

For the fecal metabolism, 3,727 metabolites were detected and 1,652 metabolites were mapped with a compound ID in KEGG, in which 635 metabolites were annotated to one or more KEGG map. Cyclamic acid, FFA (18:2), D-Phenylalanine, Urobilin, FFA (16:0), Isoetharine, and Cholic acid were the top abundant metabolites in fecal samples. Fecal metabolites were mainly involved in amino acid and its metabolites, bile acids, benzene and substituted derivatives, carbohydrates and its metabolites, nucleotide and its metabolites, fatty acyl, glycerophospholipids, heterocyclic compounds, hormones and hormone-related compounds, and organic acid and its derivatives (**Supplementary Figure S2**).

For the blood metabolites, 1,538 metabolites were revealed and 711 metabolites were mapped with a KEGG compound ID, in which 286 metabolites were annotated with the KEGG map. The abundant blood metabolites were mainly involved in amino acid and its metabolomics, benzene and substituted derivatives, organic acid and its derivatives, glycerophospholipids, fatty acyl, etc. (**Supplementary Figure S3**).

We analyzed the collected phenotypes including BMI, age, sex, DM, and RPE on the gut microbiome and microbial and plasma metabolites (**Supplementary Table S1C**). The results showed that age could significantly affect the gut microbiome (*p* = 0.0344) and fecal (*p* = 0.0186) and blood metabolites (*p* = 0.0099). BMI and DM have an effect on the fecal metabolites (*p* = 0.0579 and 0.0055, respectively). RPE has a significant effect on the blood metabolites (*p* = 0.0047). We then explored the specific effects of these factors on the gut microbiota and fecal and plasma metabolites.

### 3.2 The effect of age on gut microbiome and fecal and plasma metabolites

**Figure 2:**
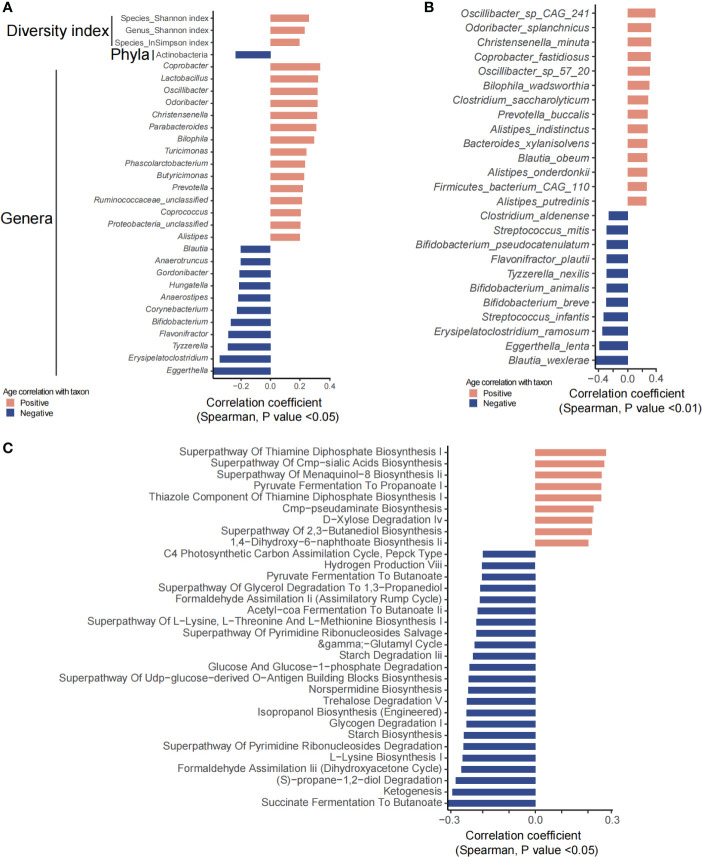
Gut microbiota and predicted functional metagenome analysis that were significantly correlated with age. **(A, B)** Show significantly correlated taxons and their relative abundance. **(C)** Predicted pathways that were significantly associated with age.

Age showed a significant effect on the gut microbiome and fecal and plasma metabolites. Phylum Actinobacteria was significantly negatively correlated with age (**Figure 2A**). Of the 29 genera that were significantly associated with age, 18 genera, including *Butyricimonas*, *Lactobacillus*, *Alistipes*, *Agrobacterium*, *Coprococcus*, *Prevotella*, *Bilophila*, and *Parabacteroides*, were positively associated with age, whereas *Bifidobacterium*, *Eggerthella*, *Erysipelatoclostridium*, *Tyzzerella*, *Flavonifractor*, *Corynebacterium*, *Anaerostipes*, *Hungatella*, *Gordonibacter*, *Anaerotruncus*, and *Blautia* were negatively correlated with age (**Figure 2A**). A total of 46 species including *Bifidobacterium dentium*, *B. adolescentis*, *Butyricimonas virosa*, *Lactobacillus mucosae*, *L. ruminis*, and *L. gasseri* were significantly positively correlated with age, while 27 species including *Bifidobacterium breve*, *B. animalis*, *B. pseudocatenulatum*, and *B. bifidum* were significantly negatively correlated with age (**Figure 2B**). Microbial metabolic pathways including pyruvate fermentation to propanoate were positively correlated with age while succinate/pyruvate/acetyl-CoA fermentation to butanoate, ketogenesis, glycogen degradation, trehalose degradation, starch biosynthesis and degradation, and glucose and glucose-1-phosphate degradation were negatively correlated with age, suggesting a decrease in the capability of butyrate production but an increase in that of propanoate (**Figure 2C**).

**Figure 3:**
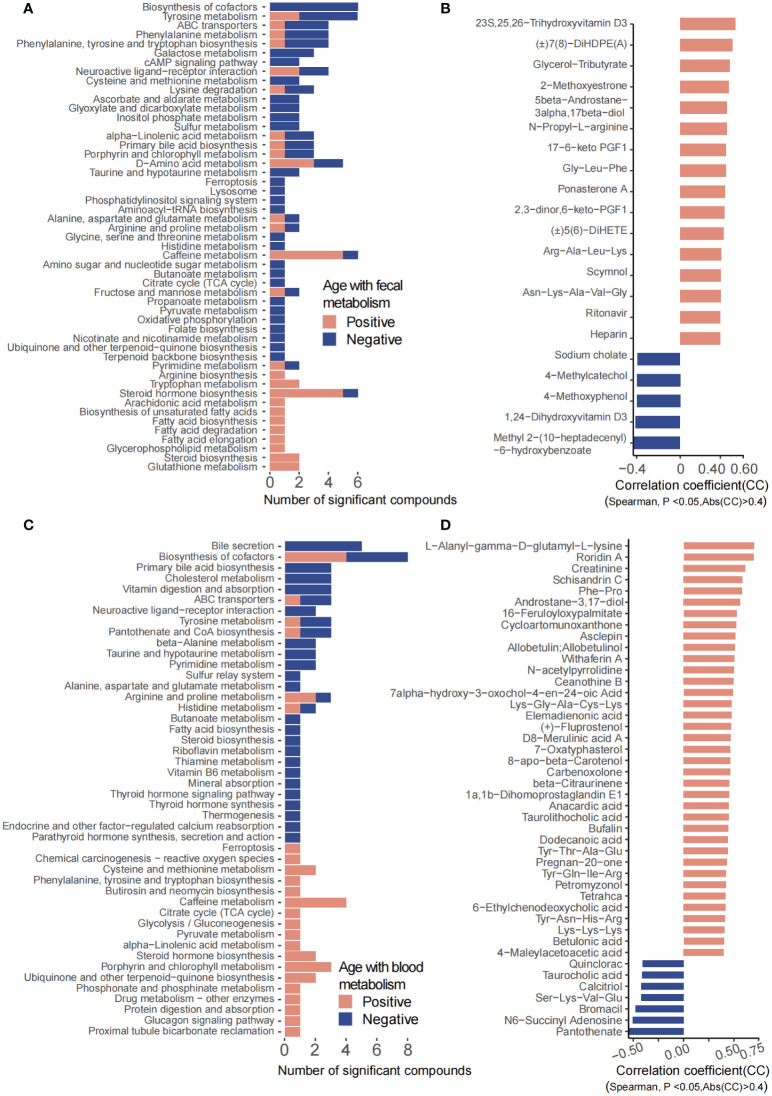
Fecal and plasma metabolites that significantly correlated with age. **(A)** KEGG pathways involved for the fecal metabolites. **(B)** Fecal metabolites that were significantly correlated with age. **(C)** KEGG pathways involved for the plasma metabolites. **(D)** Plasma metabolites that were significantly correlated with age.

Fecal metabolites showed that 534 metabolites were significantly correlated with age, in which 88 metabolites were annotated with KEGG pathways. A total of 70 metabolites including hormones and hormone-related compounds, ketones, oxidized lipids, and small peptide were significantly positively associated with age, while 18 metabolites including 1-hydroxy vitamin D3, atrolactic acid, 3-hydroxypyruvic acid, myoinositol, succinic acid, 3-indolebutyric acid, 2-(4-hydroxyphenyl) propionate, and 3-(3-hydroxyphenyl) propionate were significantly negatively associated with age (**Figure 3B**). Consistently, KEGG pathways including D-amino acid metabolism, caffeine metabolism, arginine biosynthesis, tryptophan metabolism, steroid hormone biosynthesis, arachidonic acid metabolism, biosynthesis of unsaturated fatty acids, fatty acid biosynthesis/degradation/elongation, glycerophospholipid metabolism, steroid biosynthesis, and glutathione metabolism were positively associated with age, with the above abilities of the gut microbiota increasing along with age. However, biosynthesis of cofactors, tyrosine metabolism, galactose metabolism, cAMP signaling pathway, primary bile acid biosynthesis, ascorbate and aldarate metabolism, glyoxylate and dicarboxylate metabolism, inositol phosphate metabolism, sulfur metabolism, TCA cycle, propanoate metabolism, pyruvate metabolism, oxidative phosphorylation, folate biosynthesis, nicotinate and nicotinamide metabolism, and lysosome, among others, were significantly negatively associated with age, suggesting a decrease in the energy and nutrient metabolism of the gut microbiota along with age (**Figure 3A**).

For the plasma metabolites, we have identified 97 significantly age-related metabolites, of which 52 metabolites were annotated with KEGG pathways (**Figure 3D**). Arginine and proline metabolism; cysteine and methionine metabolism; phenylalanine, tyrosine, and tryptophan biosynthesis; caffeine metabolism; TCA cycle; glycolysis/gluconeogenesis; pyruvate metabolism; steroid hormone biosynthesis; phosphonate and phosphinate metabolism; protein digestion and absorption; and glucagon signaling pathway, among others, were significantly increased along with age. In contrast, bile secretion; primary bile acid biosynthesis; cholesterol metabolism; vitamin digestion and absorption; ABC transporters; neuroactive ligand–receptor interaction; tyrosine metabolism; pantothenate and CoA biosynthesis; alanine, aspartate, and glutamate metabolism; fatty acid biosynthesis; steroid biosynthesis; riboflavin metabolism; thiamine metabolism; vitamin B6 metabolism; mineral absorption; endocrine and other factor-regulated calcium reabsorption; thyroid hormone signaling pathway; parathyroid hormone synthesis; and secretion and action, among others, were decreased with age (**Figure 3C**).

Correlation analysis was performed between the significantly age-associated gut microbiota and fecal and plasma metabolites. The age negatively related fecal metabolites were significantly positively correlated with age negatively related species; in contrast, age positively related fecal metabolites were obviously positively associated with age positively related species (**Supplementary Figure S4**). The age negatively related blood metabolites were significantly positively correlated with age negatively related species. The same results were observed for age positively correlated gut microbiota and blood metabolites (**Supplementary Figure S5**). For the fecal and blood metabolites, age negatively related fecal metabolites including sodium cholate were significantly positively associated with age negatively related blood metabolites; the same trends were observed for age positively correlated fecal and blood metabolites (**Supplementary Figure S6**).

### 3.3 Effects of BMI on the gut microbiota and metabolites

**Figure 4:**
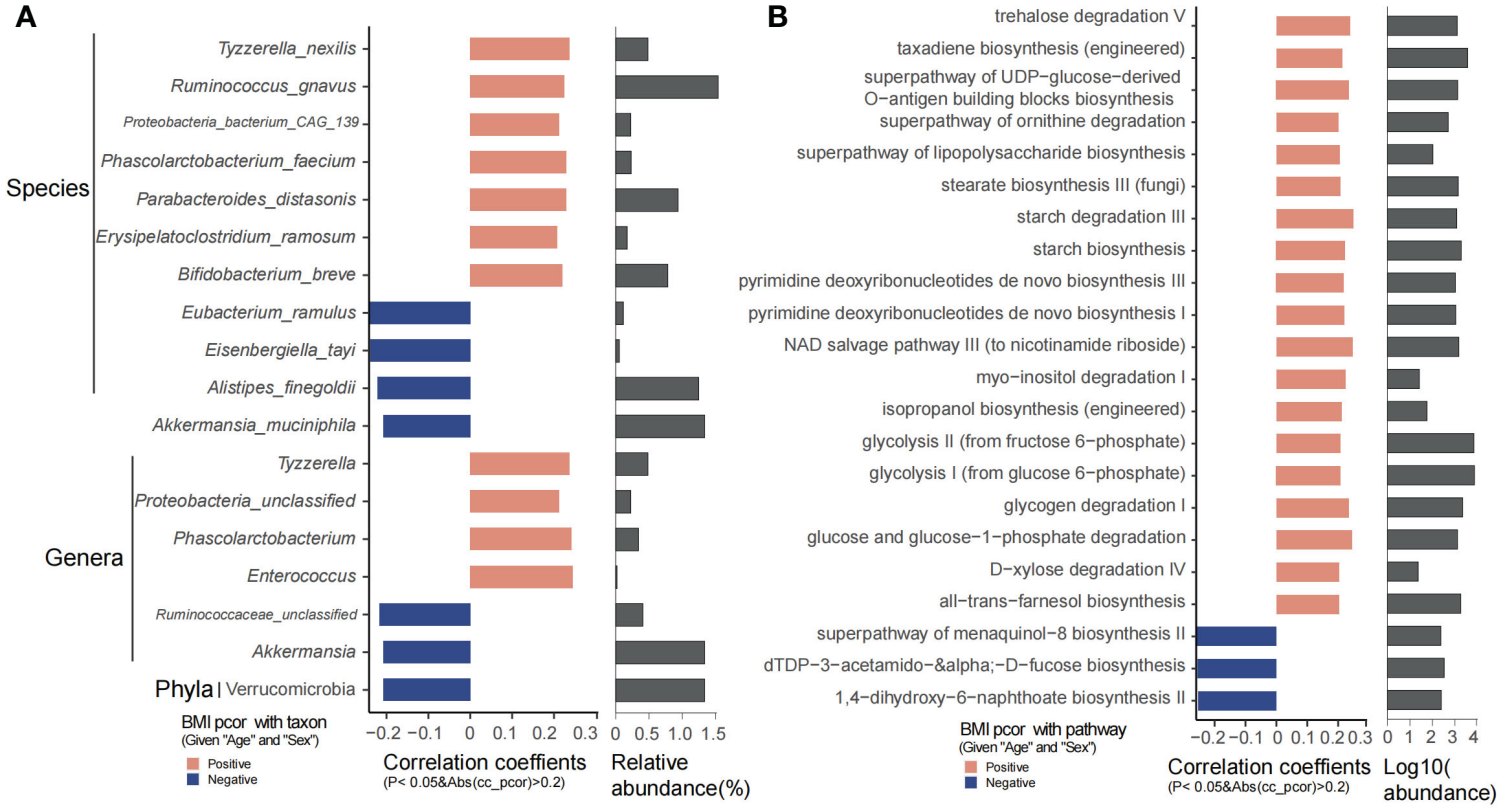
BMI significantly related taxons and predicted functional pathways. **(A)** Phyla, genera, and species that were significantly correlated with BMI. **(B)** The predicted functional pathways that were obviously correlated with BMI.

Bifidobacterium breve, Erysipelatoclostridium ramosum, Parabacteroides distasonis, Phascolarctobacterium, Phascolarctobacterium faecium, Ruminococcus gnavus, Tyzzerella, Tyzzerella nexilis, and Enterococcus were significantly positively associated with BMI, while Akkermansia, A. muciniphila, Alistipes finegoldii, Eisenbergiella tayi, and Eubacterium ramulus were obviously decreased with age (**Figure 4A**). Microbial metabolic pathways showed that trehalose degradation V, superpathway of UDP-glucose-derived O-antigen building blocks/ornithine degradation/lipopolysaccharide biosynthesis, stearate biosynthesis, starch biosynthesis and degradation, pyrimidine deoxyribonucleotides *de novo* biosynthesis, NAD salvage pathway, isopropanol biosynthesis (engineered), glucose and glucose-1-phosphate degradation, glycolysis I, glycolysis II, all-trans-farnesol biosynthesis, D-xylose degradation IV, and glycogen degradation I were significantly increased as BMI increases. However, 1,4-dihydroxy-6-naphthoate biosynthesis II, dTDP-3-acetamido-α-D-fucose biosynthesis, and superpathway of menaquinol-8 biosynthesis II were decreased along with BMI (**Figure 4B**).

For fecal metabolites, 1,091 metabolites were significantly positively associated with BMI, while 31 metabolites were significantly negatively correlated with BMI (*p* < 0.05, |correlation coefficient| > 0.2), during which 25 metabolites were annotated with the KEGG pathway (**Supplementary Figure S7A**). Endocytosis; regulation of autophagy; ABC transporters; Rap1 signaling pathway; Ras signaling pathway; arginine and proline metabolism; cysteine and methionine metabolism; glycine, serine, and threonine metabolism; lysine degradation; phenylalanine metabolism; valine, leucine, and isoleucine degradation; butirosin and neomycin biosynthesis; pantothenate and CoA biosynthesis; beta-alanine metabolism; D-amino acid metabolism; glutathione metabolism; purine metabolism; pyrimidine metabolism; and protein digestion and absorption were significantly abundant along with BMI increase, while caffeine metabolism, alpha-linolenic acid metabolism, folate biosynthesis, and biosynthesis of cofactors were decreased as BMI increases (**Supplementary Figure S7C**).

For plasma metabolites, 116 metabolites were significantly positively associated with BMI while 42 metabolites were negatively correlated with BMI (*p* < 0.05, |correlation coefficient| >0.2) (**Supplementary Figure S7B**). Of the above differential metabolites, 20 have been annotated with the KEGG pathway. Ferroptosis, ABC transporters, sulfur relay system, choline metabolism in cancer, cysteine and methionine metabolism, tyrosine metabolism, sulfur metabolism, glycerophospholipid metabolism, steroid hormone biosynthesis, nicotinate and nicotinamide metabolism, porphyrin and chlorophyll metabolism, ubiquinone and other terpenoid−quinone biosynthesis, pyrimidine metabolism, biosynthesis of cofactors, and protein digestion and absorption were significantly increased along with BMI increase, whereas phosphatidylinositol signaling system, arginine and proline metabolism, lysine degradation, phenylalanine metabolism, inositol phosphate metabolism, fatty acid biosynthesis, and D-amino acid metabolism were decreased as BMI increases (**Supplementary Figure S7D**).

Association studies between the BMI significantly related gut microbiota and fecal and plasma metabolites were performed. BMI significantly positively related fecal metabolites and blood metabolites were significantly negatively associated with BMI negatively associated species including *A. muciniphila*, *A. finegoldii*, and *E. tayi* (**Supplementary Figures S8** and **S9**). For the fecal and blood metabolites, the BMI negatively correlated fecal metabolites were significantly positively associated with BMI negatively correlated blood metabolites (**Supplementary Figure S10**).

### 3.4 Effect of delivery mode on the gut microbiota and fecal and plasma metabolites

DM including eutocia and C-section was reported to significantly affect the gut microbiota in newborn babies. In our study, we found that DM could significantly affect the microbial metabolites (*p* < 0.05). No significant differences were observed in α-diversity at both the species and genus levels (**Supplementary Figure S11A**). *Firmicutes*, *Bacteroidetes*, Actinobacteria, Proteobacteria, and Verrucomicrobia were the main phyla between the two groups (**Supplementary Figure S11B**). Genera including *Bacteroides*, *Faecalibacterium*, *Bifidobacterium*, *Roseburia*, *Eubacterium*, and *Ruminococcus*, and species including *Faecalibacterium prausnitzii*, *Bacteroides vulgatus*, *Bacteroides uniformis*, *Ruminococcus bromii*, *Eubacterium rectale*, and *Bifidobacterium pseudocatenulatum* were the main species in both groups of children (**Supplementary Figures S11C, D**). Differential analysis showed that *Anaerofustis stercorihominis*, *Pseudoflavonifractor capillosus*, *Eubacterium ventriosum*, *Streptococcus salivarius*, *Catabacter hongkongensis*, *Agathobaculum butyriciproducens*, *Alistipes inops*, *Clostridium lavalense*, and *Collinsella intestinalis* were highly abundant in C-section, while *Streptococcus thermophilus*, *S. vestibularis*, *Bifidobacterium animalis*, *Actinomyces massiliensis*, *Lactobacillus mucosae*, *L. ruminis*, *Gemella asaccharolytica*, and *Granulicatella elegans* were significantly highly abundant in eutocia (**Supplementary Figure S12A**). For the microbial metabolic pathways, phospholipases, lipid IVA biosynthesis, gondoate biosynthesis, superpathway of glycolysis and the Entner-Doudoroff pathway, L-rhamnose degradation I, L-ornithine/L-arginine biosynthesis, superpathway of putrescine biosynthesis, inosine 5-phosphate degradation, biotin biosynthesis II, and TCA cycle I were significantly enriched in C-section; however, only L-glutamate degradation was obviously enriched in eutocia (**Supplementary Figure S12B**).

**Figure 5:**
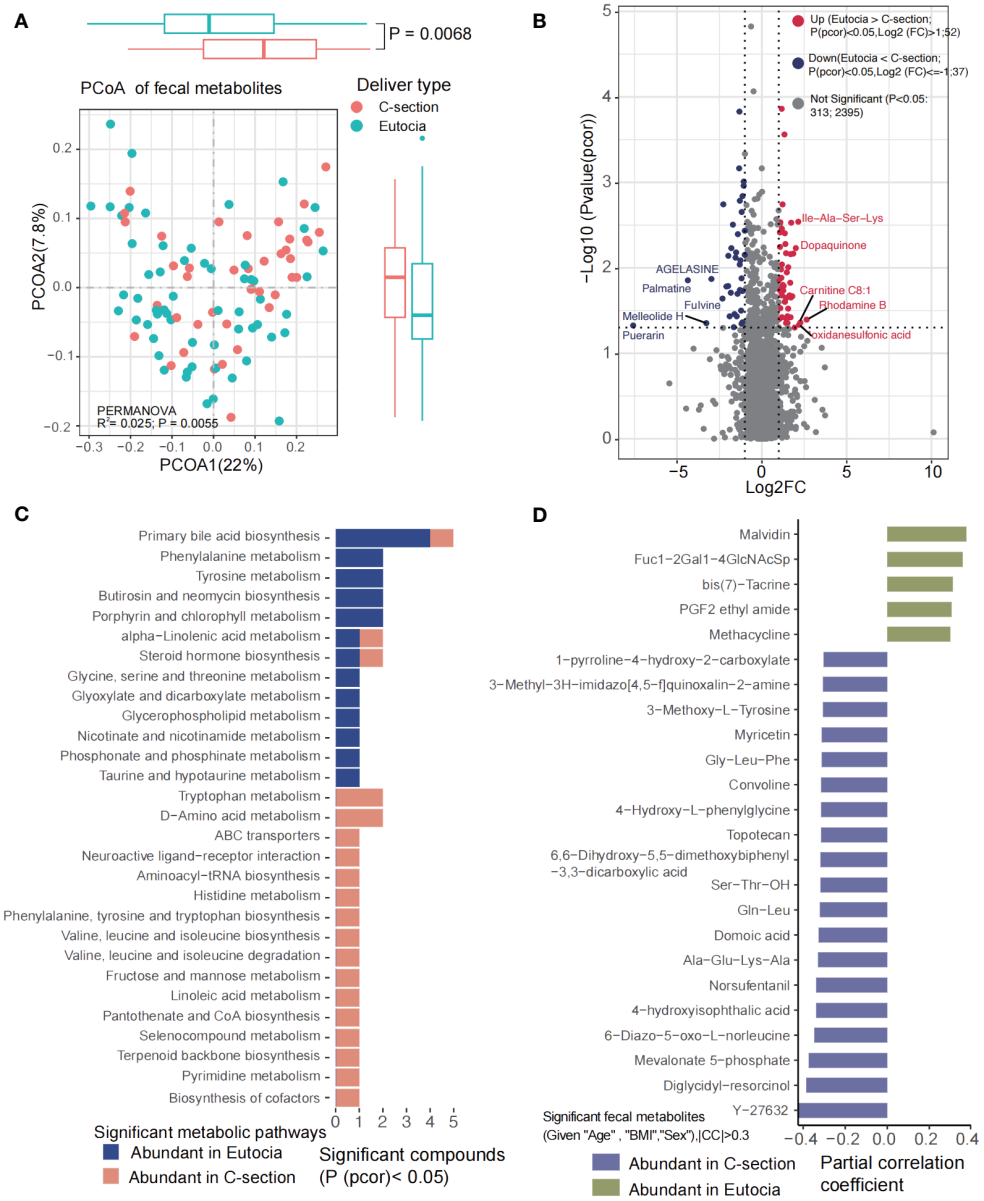
Significantly different fecal metabolites between eutocia and Cesarean section. **(A)** PCoA showed significant differences of the fecal metabolites between two groups. **(B)** Heatmap showed significantly different metabolites between two groups. **(C)** KEGG pathways involved for the fecal metabolites. **(D)** Fecal metabolites that were significantly different between two groups.

No significant differences were observed for the plasma metabolites between two groups; however, the gut microbial metabolites were significantly different between eutocia and C-section (**Figure 5A**). A total of 52 metabolites including malvidin, PGF2 ethyl amide, and methacycline were significantly enriched in eutocia while 37 metabolites including diglycidyl-resorcinol and mevalonate 5-phosphate were obviously abundant in C-section (**Figures 5B, C**). KEGG pathways including primary bile acid biosynthesis; phenylalanine and tyrosine metabolism; glycine, serine, and threonine metabolism; glycerophospholipid metabolism; nicotinate and nicotinamide metabolism; phosphonate and phosphinate metabolism; and taurine and hypotaurine metabolism were significantly abundant in eutocia (**Figure 5D**). However, tryptophan metabolism; D-amino acid metabolism; ABC transporters; neuroactive ligand–receptor interaction; histidine metabolism; phenylalanine, tyrosine, and tryptophan biosynthesis; valine, leucine, and isoleucine biosynthesis and degradation; fructose and mannose metabolism; linoleic acid metabolism; pantothenate and CoA biosynthesis; pyrimidine metabolism; and biosynthesis of cofactors were significantly abundant in C-section (**Figure 5D**). Correlation analysis between the significantly different microbial metabolites and gut microbiota between eutocia and C-section showed that eutocia-abundant species were positively associated with eutocia-abundant fecal metabolites (**Supplementary Figure S13**).

### 3.5 Physical exercise affects the gut microbiota and fecal and plasma metabolites

**Figure 6:**
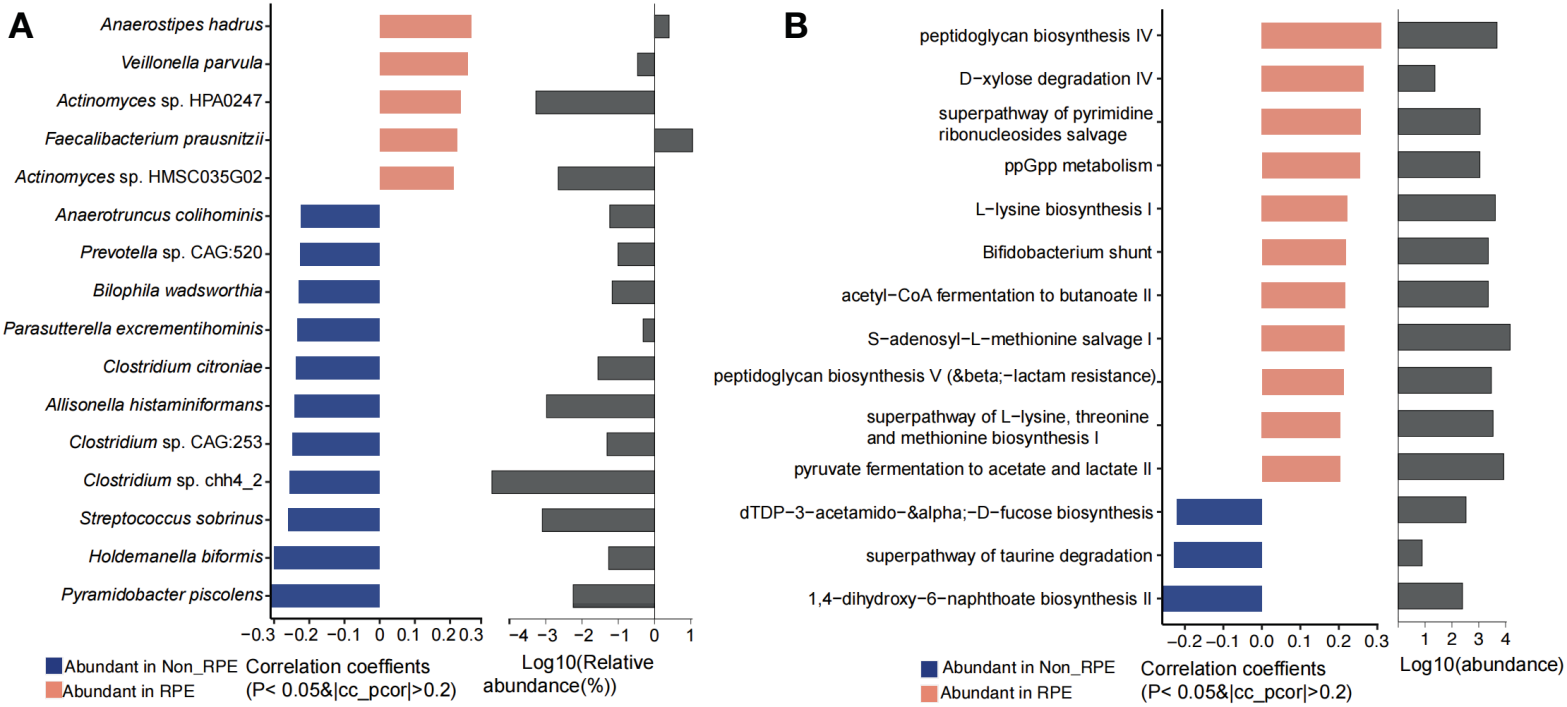
Significantly different gut microbiota and functional metabolites between with and without physical exercise. **(A)** Significantly different genera and species and their relative abundance. **(B)** Significantly different functional pathways and their relative abundance.

Physical exercise has a significant effect on the gut microbiota and metabolites. In our study, we have revealed that RPE could significantly affect the blood metabolites (*p* = 0.0047). Differential analysis of the gut microbiota showed that *Dorea*, *Faecalibacterium*, *Veillonella*, *Anaerostipes*, *F. prausnitzii*, *Veillonella parvula*, and *Anaerostipes hadrus* were highly abundant in children with RPE. However, *Pyramidobacter piscolens*, *Holdemanella biformis*, *Streptococcus sobrinus*, *Clostridium* spp., *Allisonella histaminiformans*, *Parasutterella excrementihominis*, *Bilophila wadsworthia*, and *Anaerotruncus colihominis* were evidently increased in children who have no RPE (**Figure 6A**). The highly abundant phylum Synergistetes, genera *Faecalibacterium* and *Anaerostipes*, and species *F. prausnitzi* and *Anaerostipes hadrus* were significantly enriched in children with RPE (**Supplementary Figure S14**). Microbial metabolic pathways including pyruvate fermentation to acetate and lactate II, superpathway of L-lysine, threonine and methionine biosynthesis I, peptidoglycan biosynthesis, S-adenosyl-L-methionine salvage I, acetyl-CoA fermentation to butanoate, Bifidobacterium shunt, L-lysine biosynthesis I, and peptidoglycan biosynthesis IV were significantly abundant in children with RPE. Interestingly, 1,4-dihydroxy-6-naphthoate biosynthesis II and superpathway of taurine degradation were significantly enriched in children without RPE (**Figure 6B**).

A total of 39 microbial metabolites were significantly higher in children with RPE while 33 metabolites were obviously enriched in children without RPE (**Supplementary Figures S7A, B**). Eighteen fecal metabolites were annotated with KEGG pathways, in which ABC transporters; galactose metabolism; taurine and hypotaurine metabolism; arginine and proline metabolism; glycine, serine, and threonine metabolism; phenylalanine, tyrosine, and tryptophan biosynthesis; caffeine metabolism; amino sugar and nucleotide sugar metabolism; fructose and mannose metabolism; primary bile acid biosynthesis; D-amino acid metabolism; and glutathione metabolism were significantly enriched in children with RPE. In contrast, alanine, aspartate, and glutamate metabolism; lysine degradation; valine, leucine, and isoleucine biosynthesis and degradation; pentose phosphate pathway; biosynthesis of unsaturated fatty acids; linoleic acid metabolism; and vitamin B6 metabolism were obviously higher in children without RPE (**Supplementary Figure S7C**).

**Figure 7:**
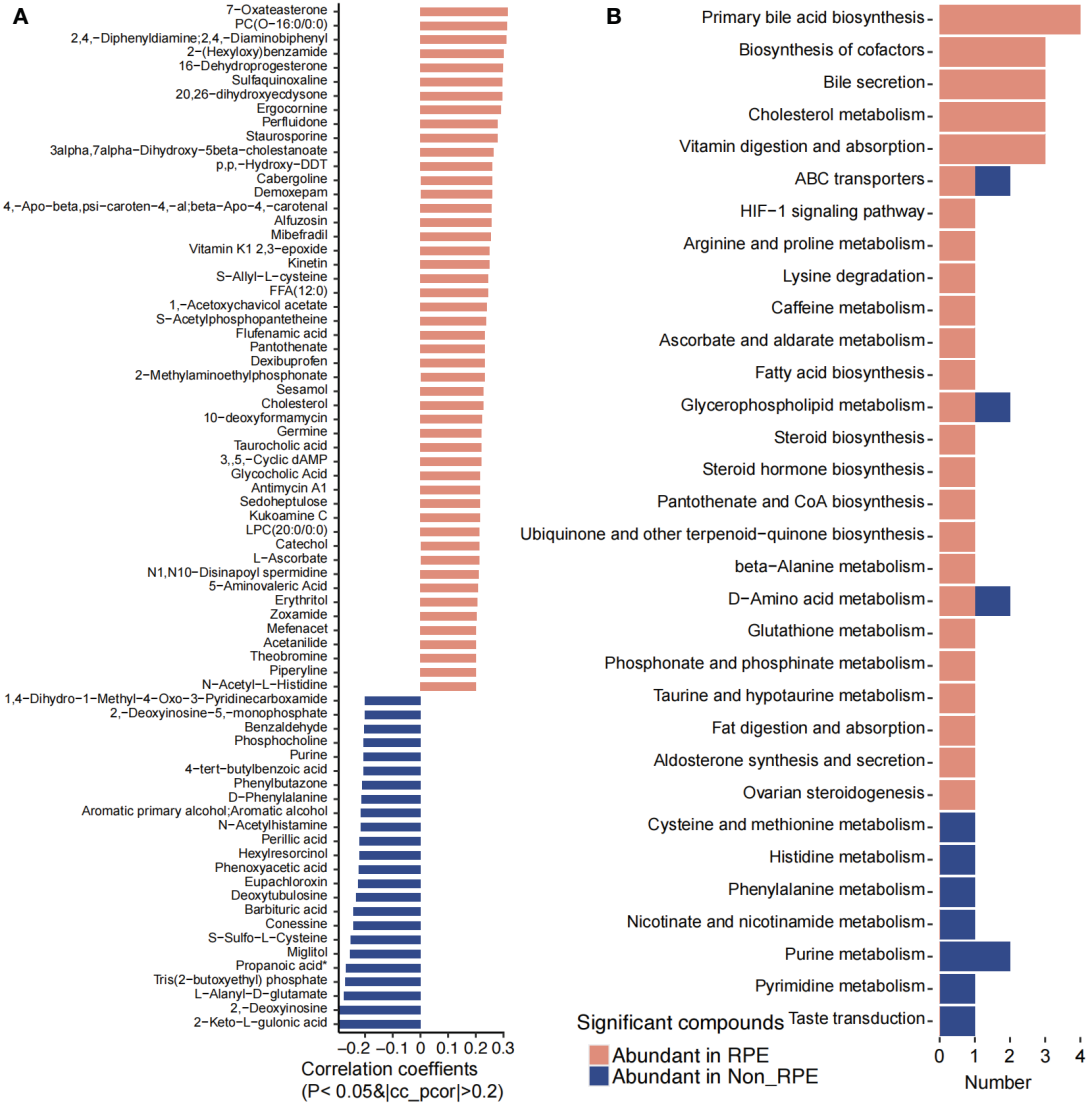
Significantly different blood metabolites between with and without regular physical exercise. **(A)** The blood metabolites that were significantly abundant in children with and without regular physical exercise. **(B)** The KEGG pathways that were involved for the significantly different blood metabolites.

A total of 167 plasma metabolites were significantly higher in children with RPE while 29 plasma metabolites were obviously enriched in children without RPE (**Figure 7B**). Eighteen metabolites were annotated with KEGG pathways. Primary bile acid biosynthesis, bile secretion, biosynthesis of cofactors, cholesterol metabolism, vitamin digestion and absorption, HIF-1 signaling pathway, arginine and proline metabolism, lysine degradation, caffeine metabolism, fatty acid biosynthesis, steroid hormone biosynthesis, pantothenate and CoA biosynthesis, beta-alanine metabolism, glutathione metabolism, fat digestion and absorption, and ovarian steroidogenesis were highly abundant in children with RPE. Cysteine and methionine metabolism, histidine metabolism, phenylalanine metabolism, nicotinate and nicotinamide metabolism, purine metabolism, pyrimidine metabolism, and taste transduction were obviously higher in children without RPE (**Figure 7A**).

The correlation of the significantly different gut microbiota and fecal and blood metabolites between the children with and without RPE was analyzed. The RPE-abundant gut species were positively correlated with the RPE-abundant fecal metabolites (**Supplementary Figure S15**) and blood metabolites (**Supplementary Figure S16**). Metabolite correlation showed that the RPE-abundant fecal metabolites were positively correlated with those in blood (**Supplementary Figure S17**).

## 4 Discussion

Gut microbiota exerts considerable effects on the child’s physical and mental development. This study was performed on 100 outwardly healthy children from Northwest China to characterize their gut microbiota and microbial and plasma metabolites. Interestingly, we have revealed that age, BMI, delivery type, and RPE could significantly alter gut microbiota and metabolome in children from Northwest China.

Age was reported to affect the gut microbiota in early-life children. Recently, the gut microbiome and metabolome in children with autoimmune diseases including systemic lupus erythematosus (Wen et al., 2021b), Henoch–Schönlein Purpura (Wen et al., 2021a), new-onset type 1 diabetes (Yuan et al., 2022), and diarrhea infected by diarrheagenic *E. coli* (Gallardo et al., 2020) and full-scale intelligence (van de Wouw et al., 2023) were investigated. In our study, several beneficial species of *Bifidobacteria* such as *B. pseudocatenulatum*, *B. breve*, and *B. animalis*; *Blautia wexlerae* (Hosomi et al., 2022); and *Flavonifractor plautii* (Luo et al., 2023), as well as some emerging pathogens such as *Eggerthella lenta* (Dong et al., 2022), *E. ramosum* (Milosavljevic et al., 2021; Iadsee et al., 2023), and *Clostridium aldenense* (Warren et al., 2006), were significantly decreased with age in our observation. Interestingly, *Lactobacillus* spp. and *Prevotella* spp.; SCFA producer *Butyricimonas* and *Odoribacter splanchnicus* (Hiippala et al., 2020); beneficial species *Christensenella minuta* (Singh and Natraj, 2021) and *Bilophila wadsworthia* (Natividad et al., 2018); anti-infectious *Blautia obeum* (Hatziioanou et al., 2017); carbohydrate metabolism promoting species *Alistipes indistinctus*, *Alistipes onderdonkii*, and *Alistipes putredinis* (Takeuchi et al., 2023); gut nicotine degrading bacteria *Bacteroides xylanisolvens* (Chen et al., 2022); saccharolytic and therapeutic drug bioaccumulation-promoting species *Clostridium saccharolyticum* (William and Murray, 1982); and commensal species *Coprobacter fastidiosus* (Shkoporov et al., 2013) were evidently increased with age in our study. Consistently, metabolic pathways including pyruvate/succinate/acetyl-CoA fermentation to butanoate, starch biosynthesis and degradation, glucose and glucose-1-phosphate degradation, and glycogen and trehalose degradation were significantly decreased with age; however, pyruvate fermentation to propanoate and D-xylose degradation were significantly increased along with age. For fecal metabolome, the gut microbial ability of fatty acid and glycerophospholipid metabolism and biosynthesis of unsaturated fatty acids, caffeine metabolism, tryptophan metabolism, and steroid and steroid hormone biosynthesis were significantly enhanced along with age. Interestingly, galactose metabolism; biosynthesis of cofactors; tyrosine metabolism; ABC transporters; phenylalanine, tyrosine, and tryptophan biosynthesis; cAMP signaling pathway; and taurine and hypotaurine metabolism were decreased along with age. In blood, caffeine metabolism, steroid hormone biosynthesis, and cysteine and methionine metabolism were increased along with age, while bile secretion, primary bile acid biosynthesis, cholesterol metabolism, vitamin digestion and absorption, and neuroactive ligand–receptor interaction were significantly decreased along with age. These results suggested that functional changes in gut microbiota were consistent with changes in their food, as microbiota participating in amino acid and vitamin metabolism were enriched in young children, whereas microbiota involved in lipid metabolism increased with age.

BMI is used to screen for potential weight and health-related issues. Here, we calculated BMI using the Child & Teen BMI Calculator (BMI Calculator Child and Teen | Healthy Weight | CDC). Lower BMI was associated with a decrease in fecal Tenericutes and an increase in Actinobacteria (Frenn et al., 2023). In our study, we found significant differences in gut microbiota bacterial composition and function along with BMI. *Bifidobacterium breve*, *E. ramosum*, *Parabacteroides distasonis*, *Phascolarctobacterium faecium*, *Ruminococcus gnavus*, and *Tyzzerella nexilis* were significantly increased with BMI, while *A. muciniphila*, *A. finegoldii*, *E. tayi*, and *E. ramulus* were significantly decreased with BMI. Studies have indicated that obese-related gut microbiota were reduced in *Bifidobacterium* and α-diversity but enriched in *Lactobacillus* (Morgado et al., 2023). Functional analysis revealed that superpathway of lipopolysaccharide biosynthesis, stearate biosynthesis, and carbohydrate metabolism including trehalose degradation, starch degradation, glycolysis, glycogen degradation, glucose and glucose-1-phosphate degradation, and D-xylose degradation were obviously increased along with BMI. Accumulating evidence has demonstrated that the gut microbiome and its metabolites play a crucial role in the onset and development of obesity (Wu et al., 2021) as signaling molecules and substrates for metabolic reactions (Krautkramer et al., 2021). We observed that metabolites from sugars, lipids, and amino acids were increased in both faces and blood along with BMI, suggesting that children with higher BMI have a greater ability to metabolize nutrients in their gut microbiota.

DM is an important determinant of gut microbiota during the first 3 years of life. Studies showed that C-section could influence the activation of intestinal epithelial cells and the development of the immune system. The feces of vaginally delivered infants were reported to have higher abundance of *Bifidobacterium*, *Lactobacillus*, *Bacteroides*, and *Parabacteroides* while C-section delivered infants were more enriched in *Klebsiella*, *Enterococcus*, trans-vaccenic acid, and cis-aconitic acid (Reyman et al., 2019; Li et al., 2021) at 3 months of age. Consistent with previous studies, we have revealed that *Lactobacillus mucosae*, *L. ruminis*, *Bifidobacterium animalis*, and L-glutamate degradation were enriched in eutocia children while *Anaerofustis stercorihominis*, *Pseudoflavonifractor capillosus*, *Catabacter hongkongensis*, *Clostridium lavalense*, *Collinsella intestinalis*, lipid IVA biosynthesis, superpathway of glycolysis, L-rhamnose degradation, and TCA cycle were abundant in C-section children, suggesting higher energy metabolism in C-section children. No significant differences were observed for plasma metabolites between eutocia and C-section children in our study. However, significant differences were observed for the microbial metabolome. Small peptides including Gly−Leu−Phe, Ala−Glu−Lys−Ala, Gln−Leu, and Ser−Thr−OH were enriched in C-section children. Conformably, KEGG pathways including tryptophan metabolism, D-amino acid metabolism, ABC transporters, fructose and mannose metabolism, and biosynthesis of cofactors were the main metabolic pathways involved for these metabolites. In contrast, primary bile acid biosynthesis; phenylalanine metabolism; tyrosine metabolism; glycine, serine, and threonine metabolism; glycerophospholipid metabolism; nicotinate and nicotinamide metabolism; phosphonate and phosphinate metabolism; and taurine and hypotaurine metabolism were the major metabolic pathways participated by microbial metabolites in eutocia children, suggesting that the gut microbiota and metabolites of different DMs might have a long effect on the children’s development.

Physical activity was reported to improve the α-diversity and beneficial bacteria linked to body weight loss in children (Morgado et al., 2023). We have revealed that *F. prausnitzii*, *Veillonella parvula*, and *Anaerostipes hadrus* were significantly enriched in children with RPE, while *Pyramidobacter piscolens*, *Holdemanella biformis*, *Streptococcus sobrinus*, *Allisonella histaminiformans*, *Clostridium citroniae*, *Parasutterella excrementihominis*, *Bilophila wadsworthia*, and *Anaerotruncus colihominis* were significantly abundant in children without RPE. Tabone et al. have explored the effect of acute moderate-intensity exercise on the gut microbiota and serum and fecal metabolomes of cross-country endurance athletes, and revealed that *Romboutsia*, *Blautia*, and *Clostridium phoceensis* were modified after a controlled acute exercise session (Tabone et al., 2021). In addition, functional analysis revealed that alanine, aspartate, and glutamate metabolism, and the arginine and aminoacyl-tRNA biosynthesis pathways were the most relevant altered pathways in serum, whereas the phenylalanine, tyrosine, and tryptophan biosynthesis pathway was the most relevant pathway changed in feces (Tabone et al., 2021). Exercise was reported to reduce plasma glucose levels and relative abundance of *Proteobacteria*, but increased that of *Blautia*, *Dialister*, and *Roseburia*, and inhibited the activation of the obesity-associated NLRP3 signaling pathway in obese pediatric patients (Quiroga et al., 2020). Irregular, exhausting, or long-lasting training was found to have a negative impact on gut microbiota and impair immune response in athletes (Wegierska et al., 2022). Meanwhile, physical activity can modulate the production of key metabolites from gut microbiota (Zhang et al., 2022) to improve body metabolism and prevent diseases (Sohail et al., 2019). We have revealed that histidine metabolism; galactose metabolism; arginine and proline metabolism; glycine, serine, and threonine metabolism; phenylalanine, tyrosine, and tryptophan biosynthesis; caffeine metabolism; primary bile acid biosynthesis; D-amino acid metabolism; and glutathione metabolism were increased in children with RPE, while alanine, aspartate, and glutamate metabolism; lysine degradation; valine, leucine, and isoleucine biosynthesis and degradation; pentose phosphate pathway; and biosynthesis of unsaturated fatty acids were enriched in children without RPE, suggesting that RPE could increase the gut microbial metabolism of nutrients and energy, as well as exogenous drugs and neurostimulants such as caffeine. Consistently, pathways including primary bile acid biosynthesis, bile secretion, cholesterol metabolism, fat digestion and absorption, vitamin digestion and absorption, HIF-1 signaling pathway, arginine and proline metabolism, lysine degradation, caffeine metabolism, fatty acid biosynthesis, steroid hormone biosynthesis, D-amino acid metabolism, and glutathione metabolism were highly abundant in children with RPE while cysteine and methionine metabolism, histidine metabolism, phenylalanine metabolism, nicotinate and nicotinamide metabolism, and purine and pyrimidine metabolism were enriched in children without RPE. These results suggested that RPE could induce changes in gut microbiota and fecal and plasma metabolites, and *vice versa*, suggesting that RPE might lower inflammatory and oxidative stress to relieve metabolic disorders.

To further analyze the correlation between the gut microbiota and fecal and blood metabolites under the above affecting factors, Spearman’s rank correlation analysis was performed. Interestingly, for the continuous variables age and BMI, their positively correlated species, fecal and plasma metabolites, showed positive associations between each other. The same trends were observed for those negatively correlated ones. For those differential variables including eutocia/C-section and with/without RPE, the eutocia enriched species and fecal and blood metabolites showed positive associations between each other; the same was observed for C-section and with/without RPE. These results suggested the systematization of the gut microbiota and its metabolites with the circulating metabolites, reminding us of the importance of considering both the fecal and blood metabolites when studying the human gut microbiome because of the communications and cross-talk between microbial and plasma metabolites.

The advantages and limitations of the study are as follows: (1) The study has revealed the gut microbiome and fecal and plasma metabolites in Chinese children from Northwest China based on shotgun metagenomic sequencing and untargeted metabolomics for the first time, and fills the gap in the current lack of the related research; (2) the study has revealed the effect of various phenotypes on the gut microbiome and microbial and plasma metabolites; (3) all the subjects come from Lanzhou, Gansu province; our future research will be extended to children in other northwestern cities, rural areas, and different ethnic groups to comprehensively explore the gut microbial and metabolomic characteristics of children from Northwestern China; (4) a more detailed dietary intake should be recorded to explore the effects of different dietary patterns on the gut microbiome and microbial and plasma metabolites; and (5) the study narrows the age gap, dividing the subjects into preschool (3–6 years old) and primary school (6–12 years old) stages.

## 5 Conclusions

In conclusion, our findings identified the previously unknown characteristics and the effect of various phenotypes on the gut microbiota and microbial and circulating metabolites in children from Northwest China, providing important references and laying a foundation for future research on children’s gut microbiota and diseases in Northwest China.

## Data availability statement

The datasets presented in this study can be found in online repositories. The names of the repository/repositories and accession number(s) can be found below: https://db.cngb.org/search/project/CNP0004326/, CNP0004326.

## Ethics statement

The studies involving humans were approved by Ethics Committee of Lanzhou University Second Hospital (protocol code 2022A-221, date of approval March 2th 2022). The studies were conducted in accordance with the local legislation and institutional requirements. Written informed consent for participation in this study was provided by the participants’ legal guardians/next of kin. Written informed consent was obtained from the individual(s), and minor(s)’ legal guardian/next of kin, for the publication of any potentially identifiable images or data included in this article.

## Author contributions

YY: Conceptualization, Data curation, Formal analysis, Funding acquisition, Investigation, Writing – original draft. JC: Conceptualization, Data curation, Formal analysis, Funding acquisition, Investigation, Writing – original draft. HG: Data curation, Formal analysis, Investigation, Validation, Writing – original draft. MC: Investigation, Software, Writing – review & editing. MZ: Investigation, Software, Writing – review & editing. XX: Conceptualization, Formal analysis, Investigation, Methodology, Resources, Software, Supervision, Validation, Writing – review & editing. QW: Conceptualization, Data curation, Funding acquisition, Investigation, Methodology, Project administration, Resources, Software, Supervision, Visualization, Writing – review & editing.
