# Supplementary material for: Characterization of the gut microbiota and fecal and blood metabolomes under various factors in urban children from Northwest China
Source: Front Cell Infect Microbiol. 2024 Mar 22;14:1374544. doi: 10.3389/fcimb.2024.1374544 (PMC10995345; doi:10.3389/fcimb.2024.1374544)
Supplement: Supplementary file 1 [file DataSheet_1.pdf]

Supplementary materials for "Characterization of the gut microbiota, fecal and blood metabolomes under various factors in urban children from Northwest China"

This profile including 17 supplementary figures. The supplementary tables (Table S1, S2) were data sheet and were listed separately.

Supplementary figures:

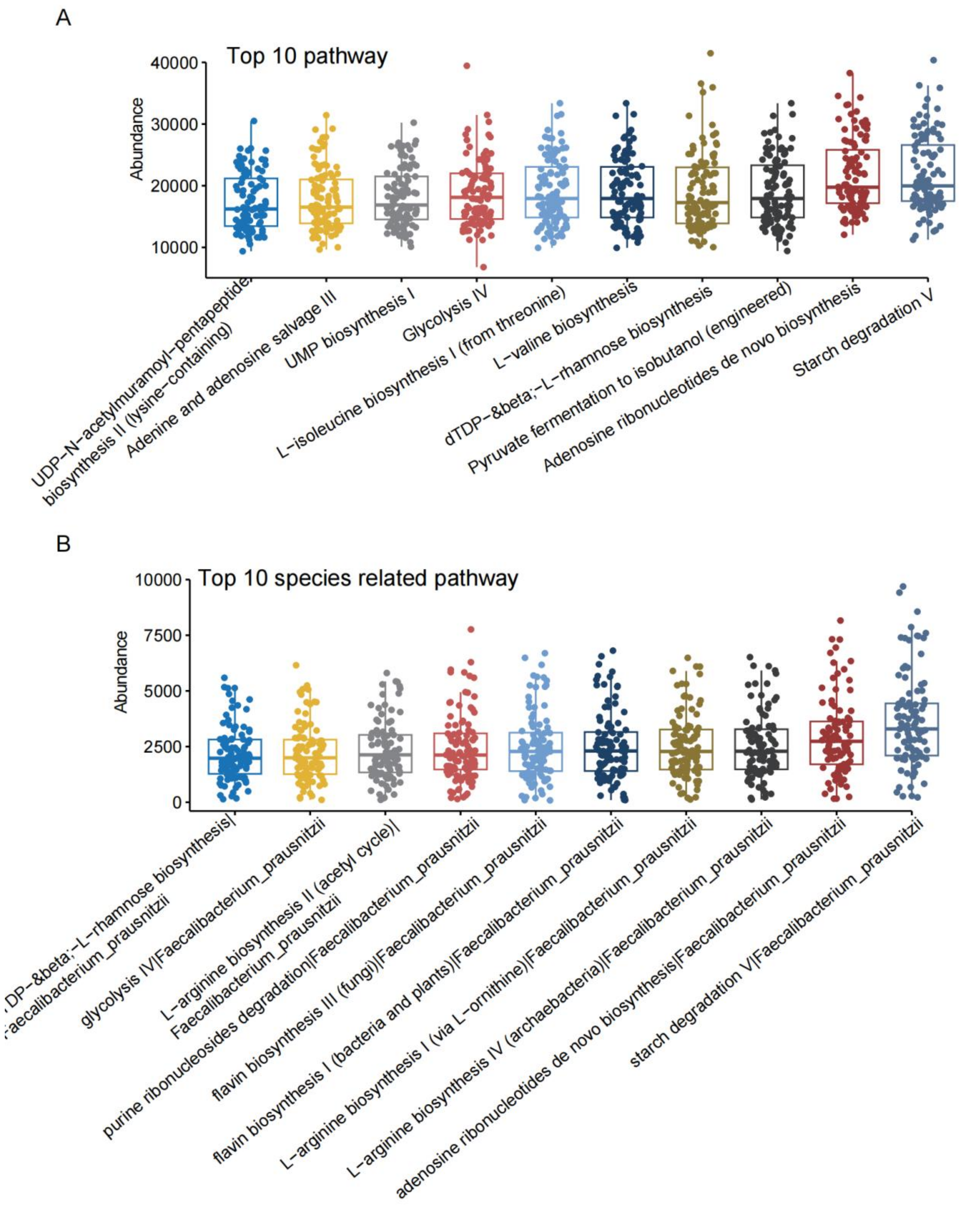

Figure S1. General analysis of the predicted functional pathways. (A) The top 10 abundant predicted functional pathways. (B) *Faecalibacterium prausnitzii* was invloved in all the top 10 abundant predicted pathways.

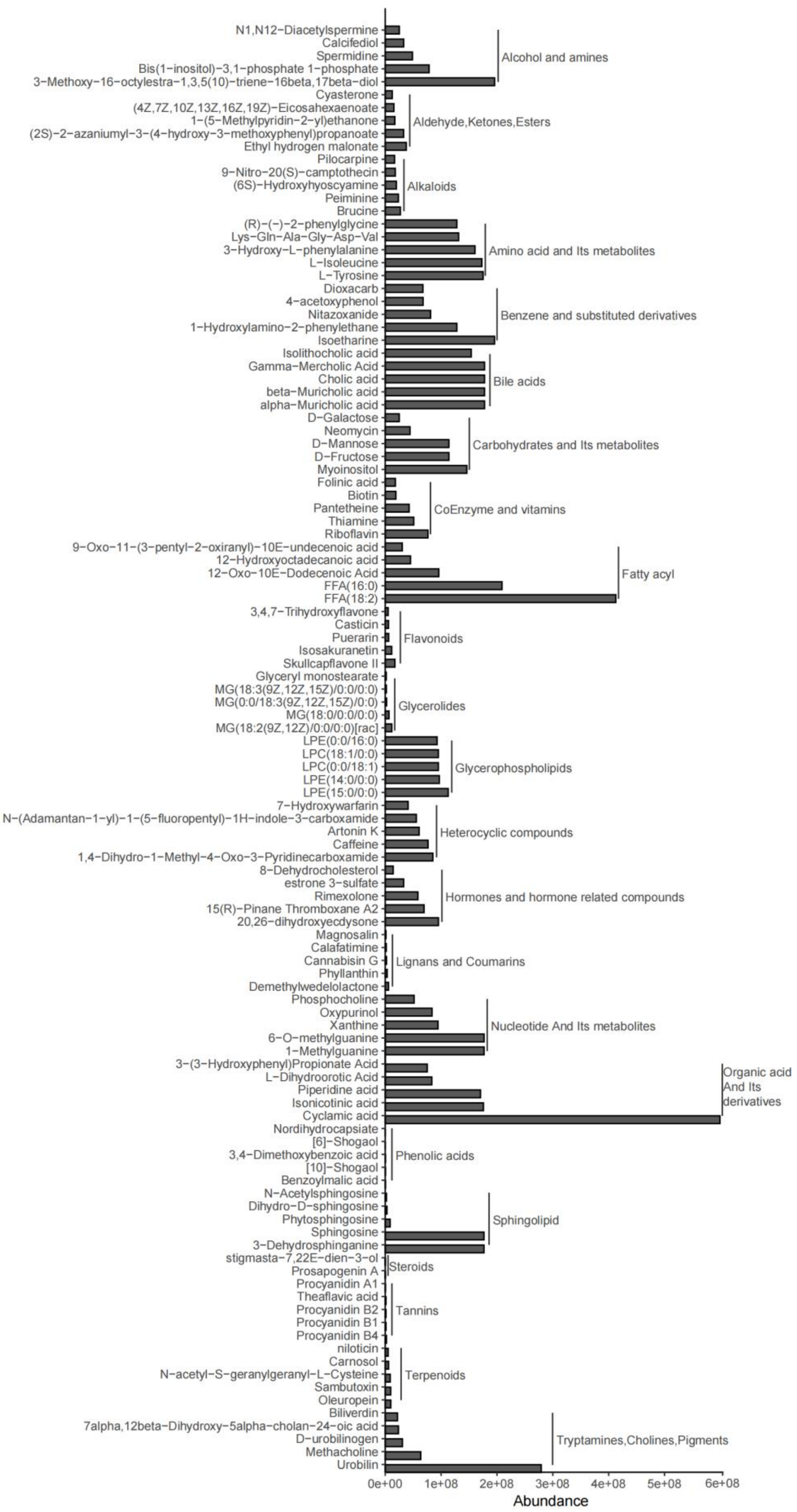

Figure S2. General analysis of the fecal metabolites, the top 5 abundant fecal metabolites in each Class 1.

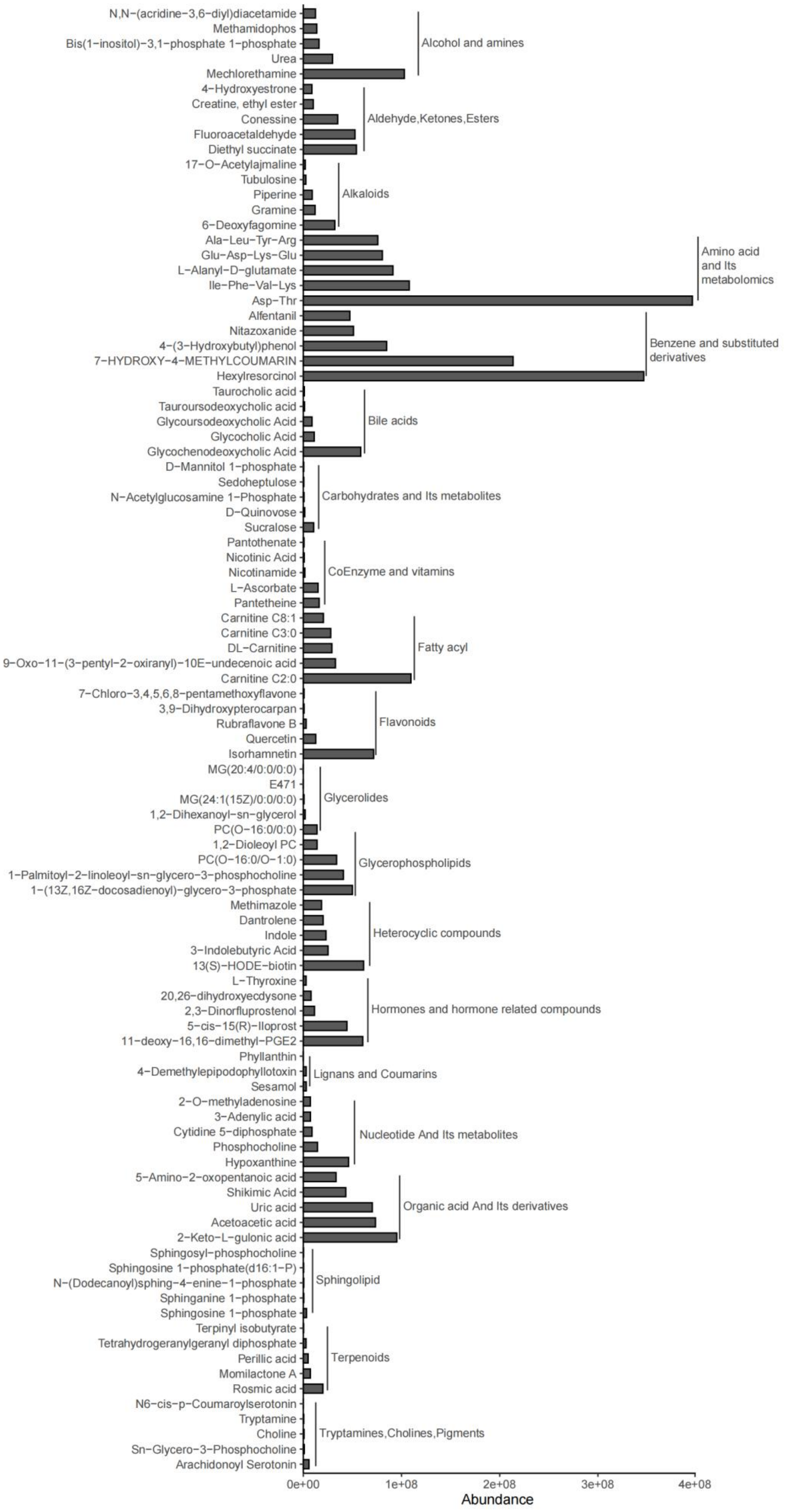

Figure S3. General analysis of the plasma metabolites, the top 5 abundant plasma metabolites in each Class 1.

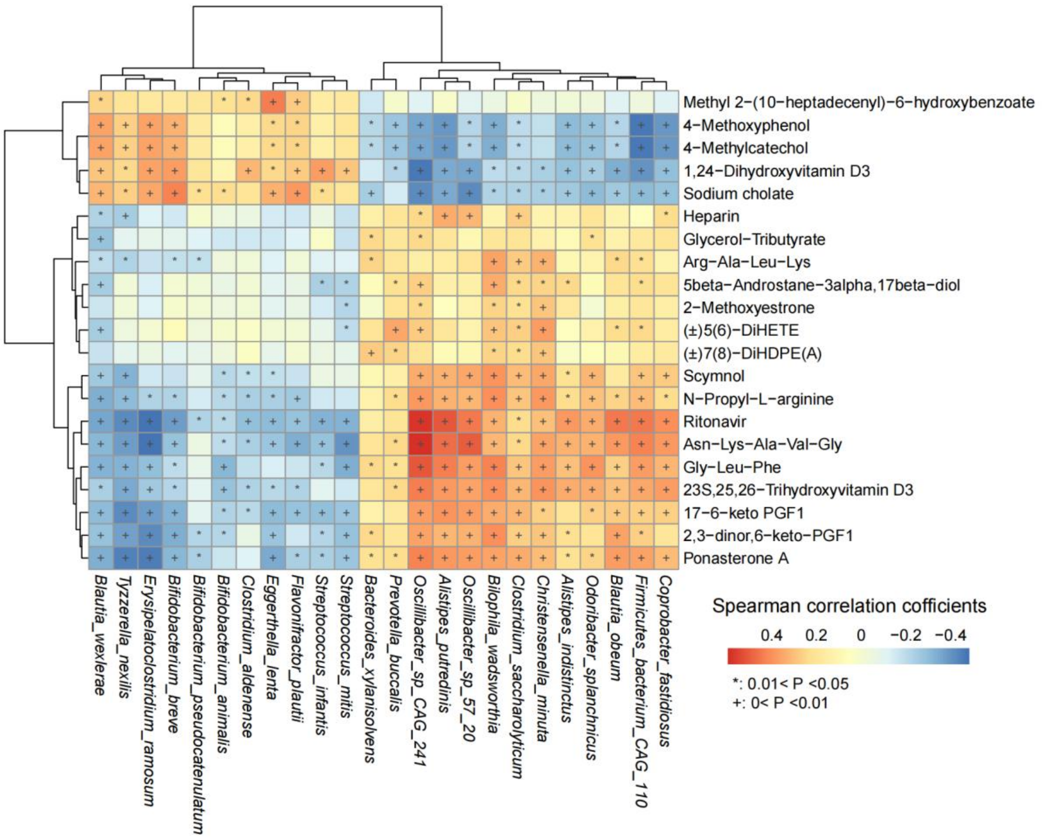

Figure S4. Correlation analysis between the significantly age related speices and fecal metabolites.

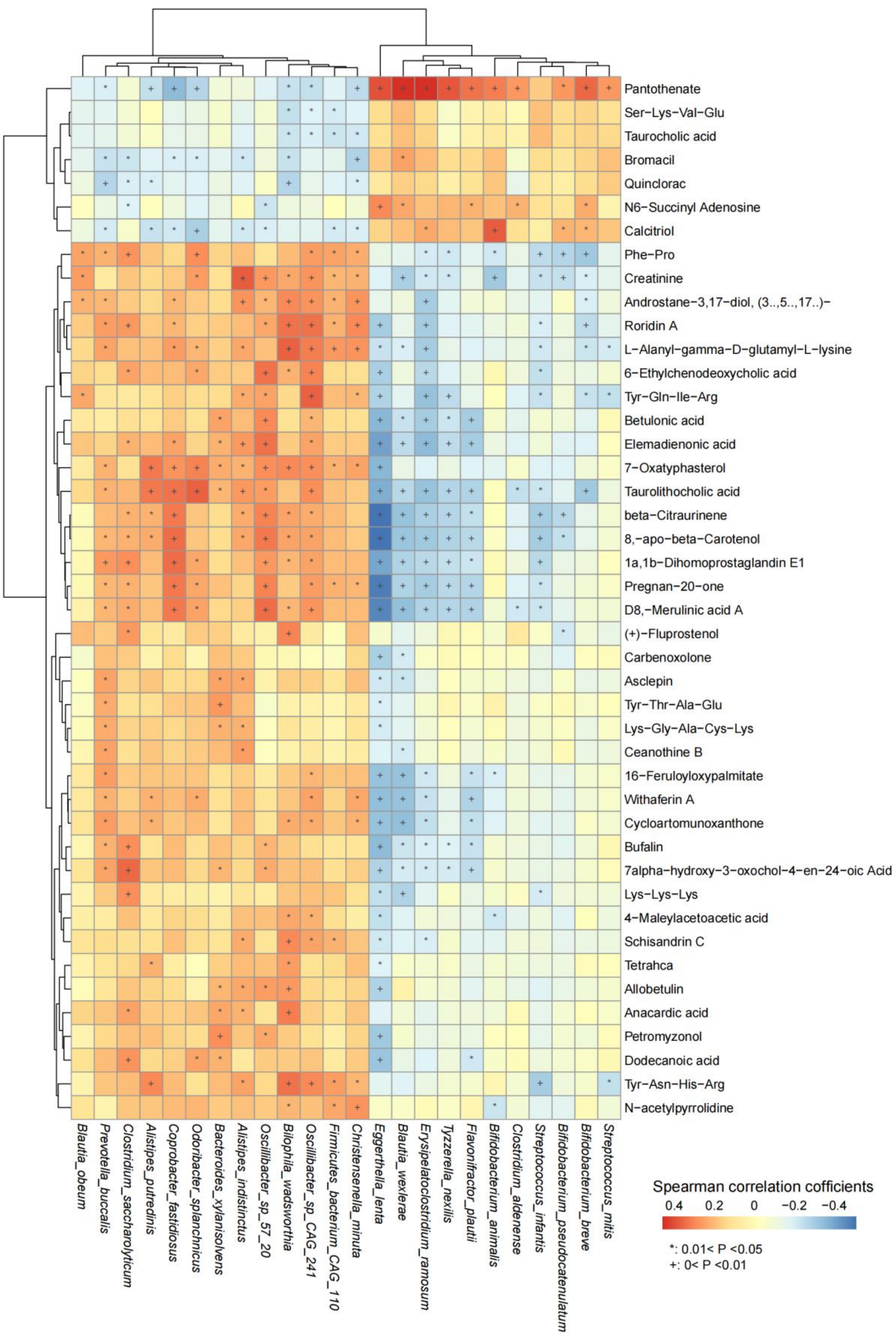

Figure S5. Correlation analysis between the significantly age related speices and blood metabolites.

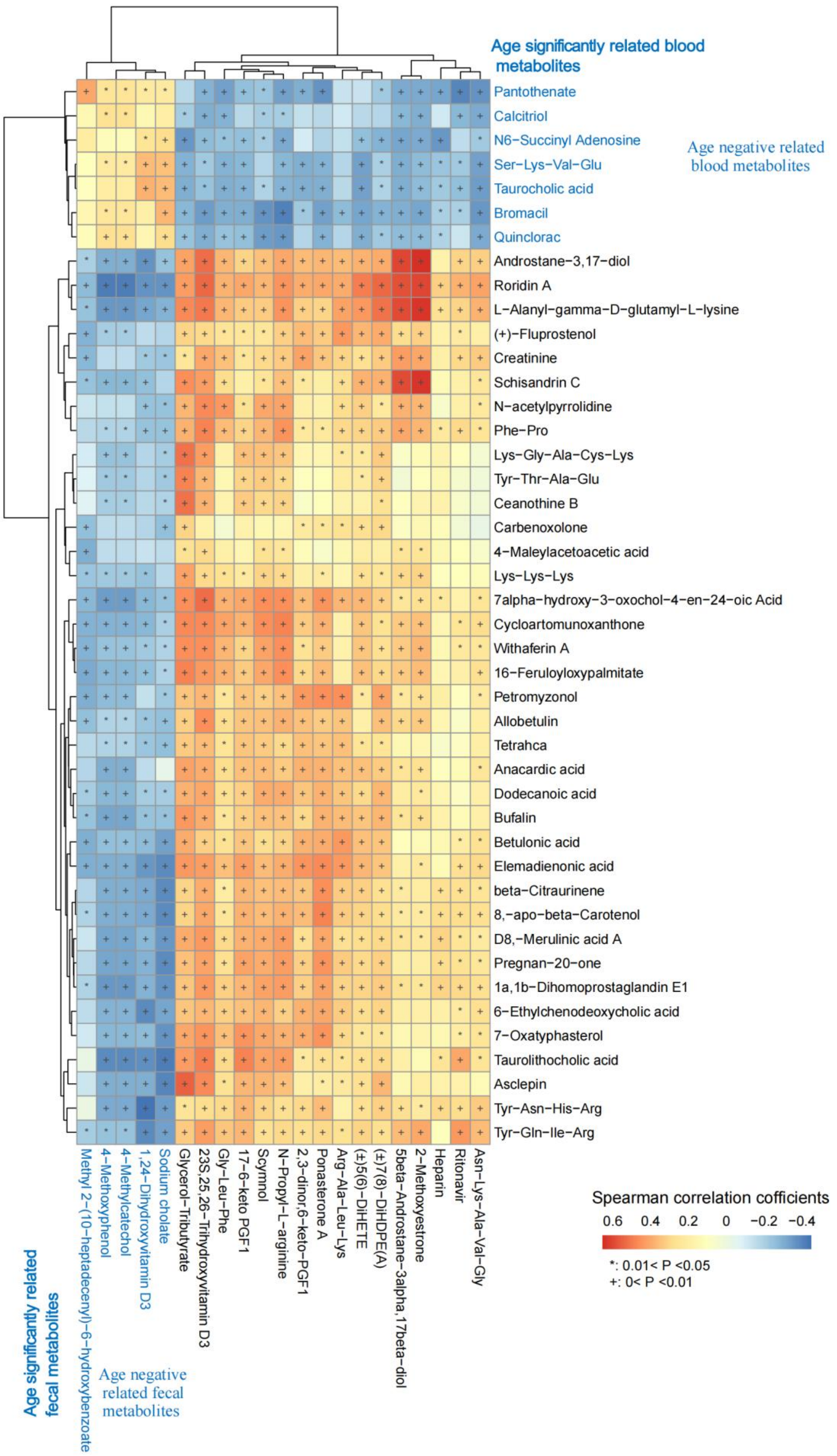

Figrue S6. Correlation analysis between the significantly age related fecal and blood metabolites.

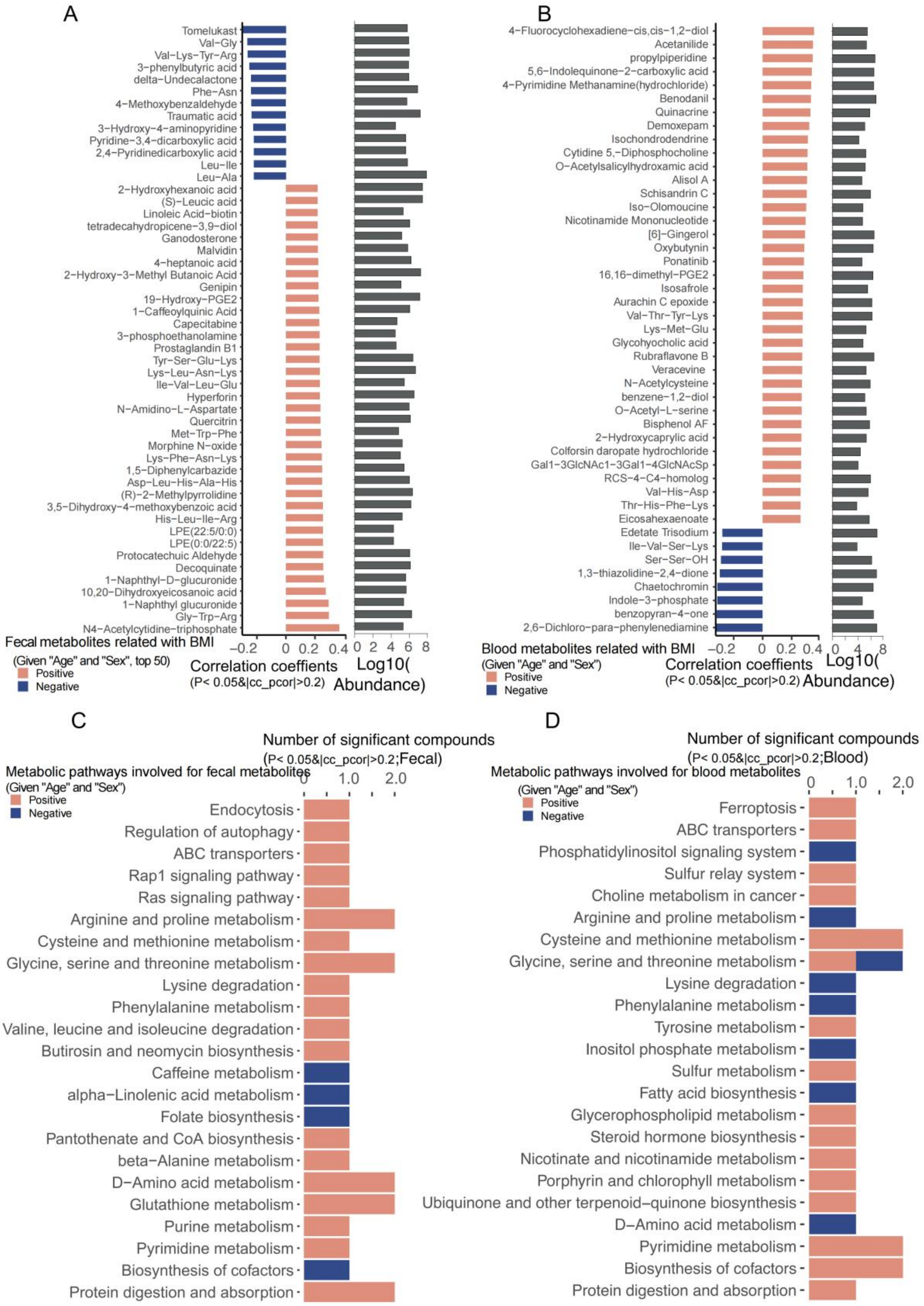

Figrue S7. Fecal and blood metabolites and the metabolic pathways that were significantly related to BMI gevin age and sex analyzed by ppcor package in R . (A) The top 50 fecal metabolites that were significantly postively and negatively correlated with BMI. (B) Blood metabolites that were significantly postively and negatively correlated with BMI. (C) KEGG metabolic pathways that were significantly involved for fecal metabolites. (D) KEGG metabolic pathways that were significantly involved for the blood metabolites. P<0.05 and |cc_pcor|>0.2 was thought to be significantly correlated.

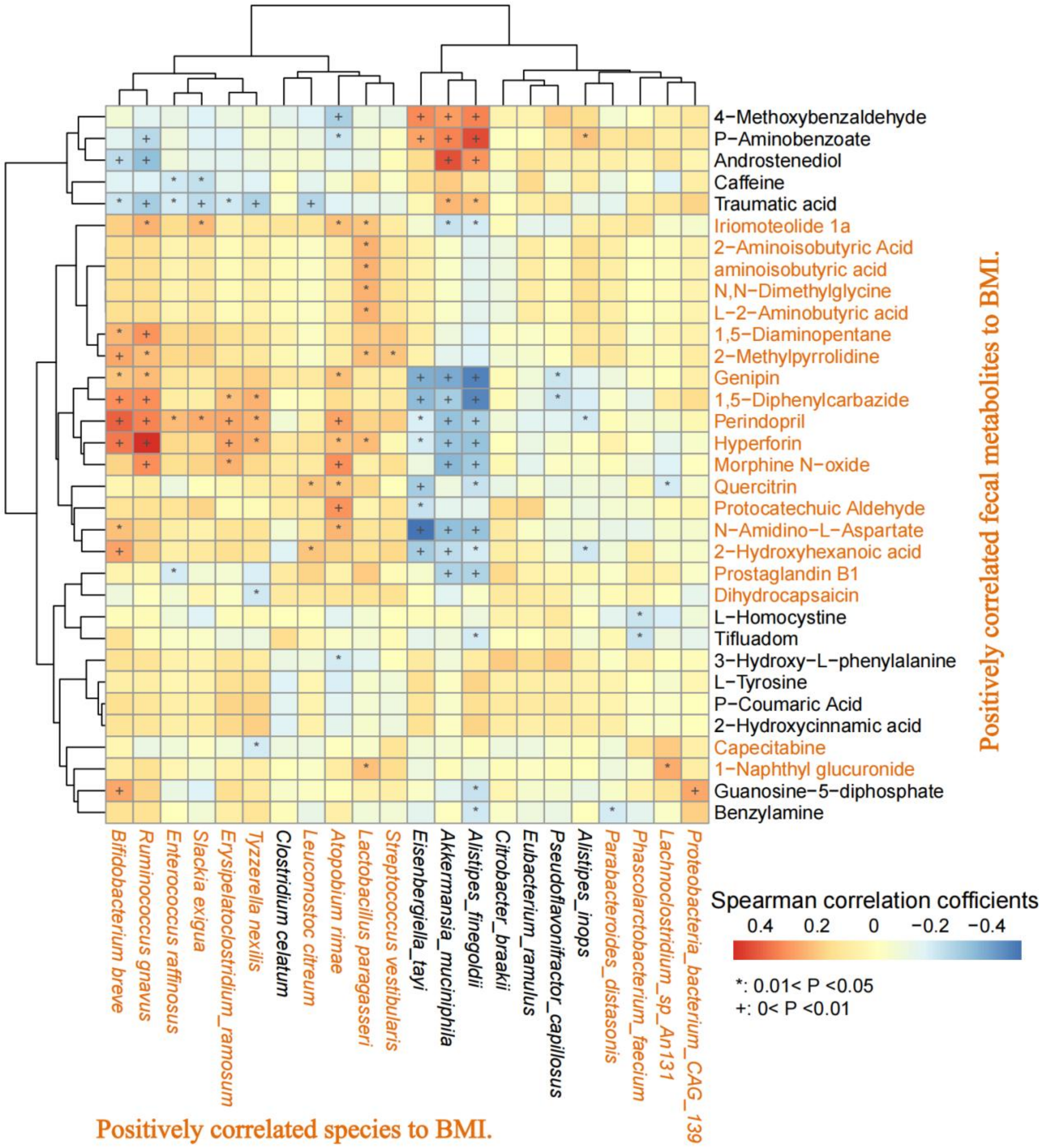

Figure S8. Correlation analysis between the BMI significantly correlated species and fecal metabolites.

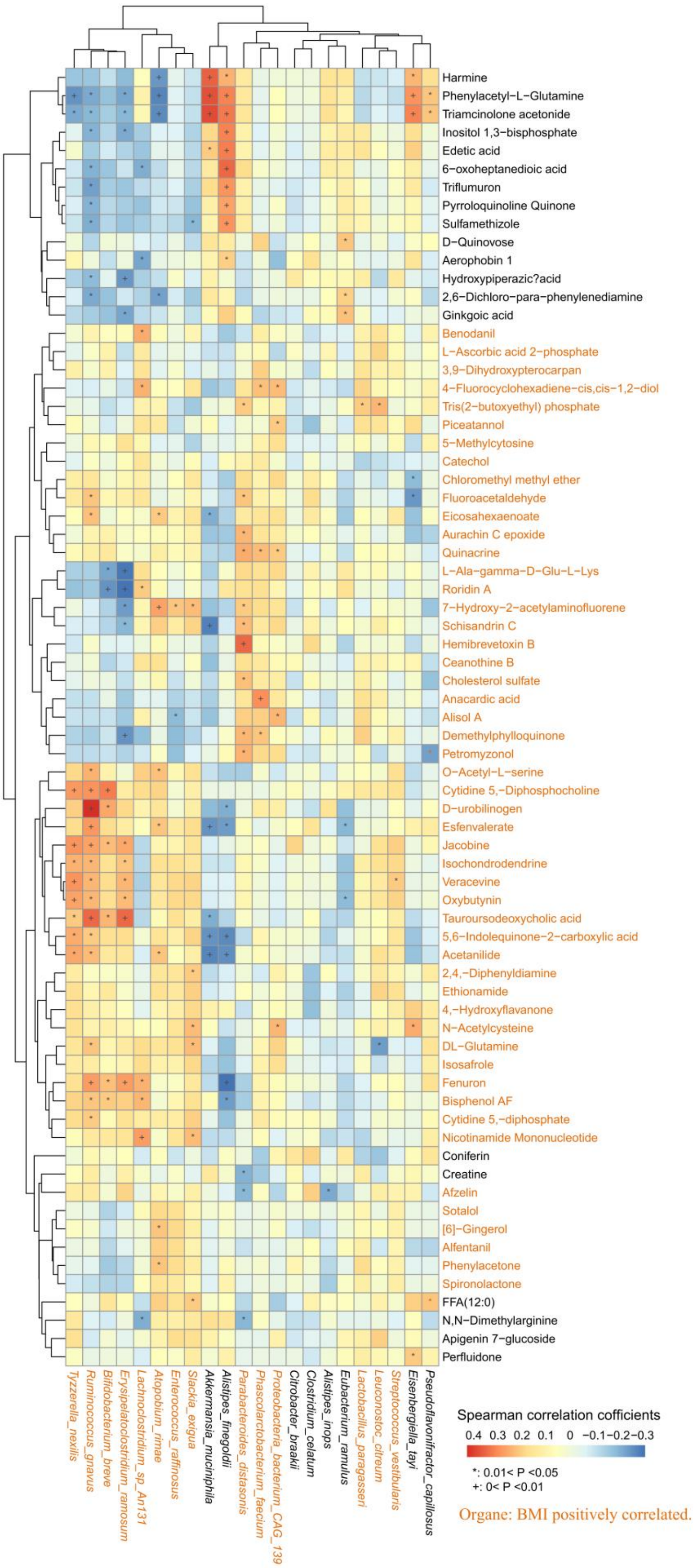

Figure S9. Correlation analysis between the BMI significantly correlated species and fecal metabolites.

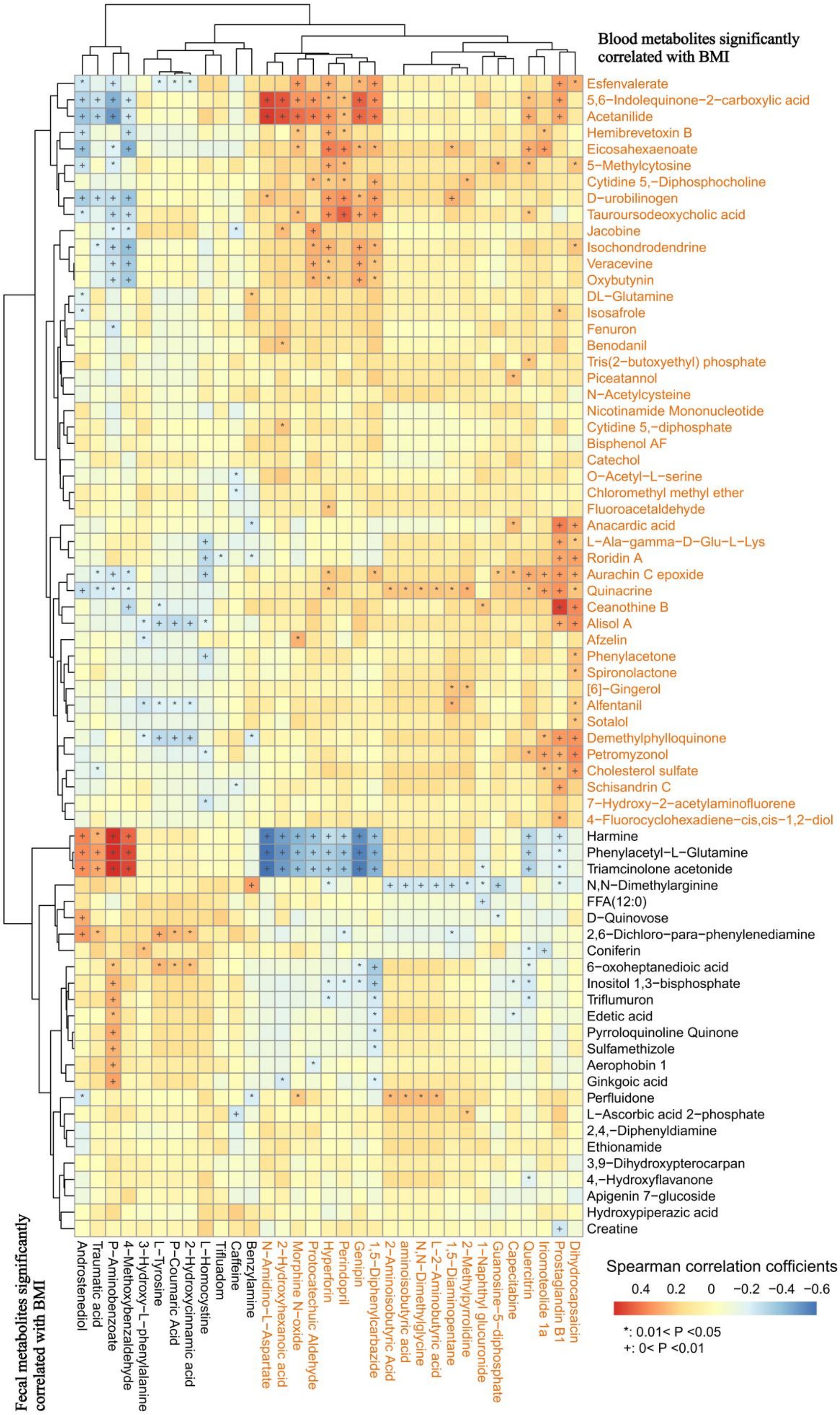

Figure S10. Correlation analysis between the BMI significantly correlated fecal and blood metabolites.

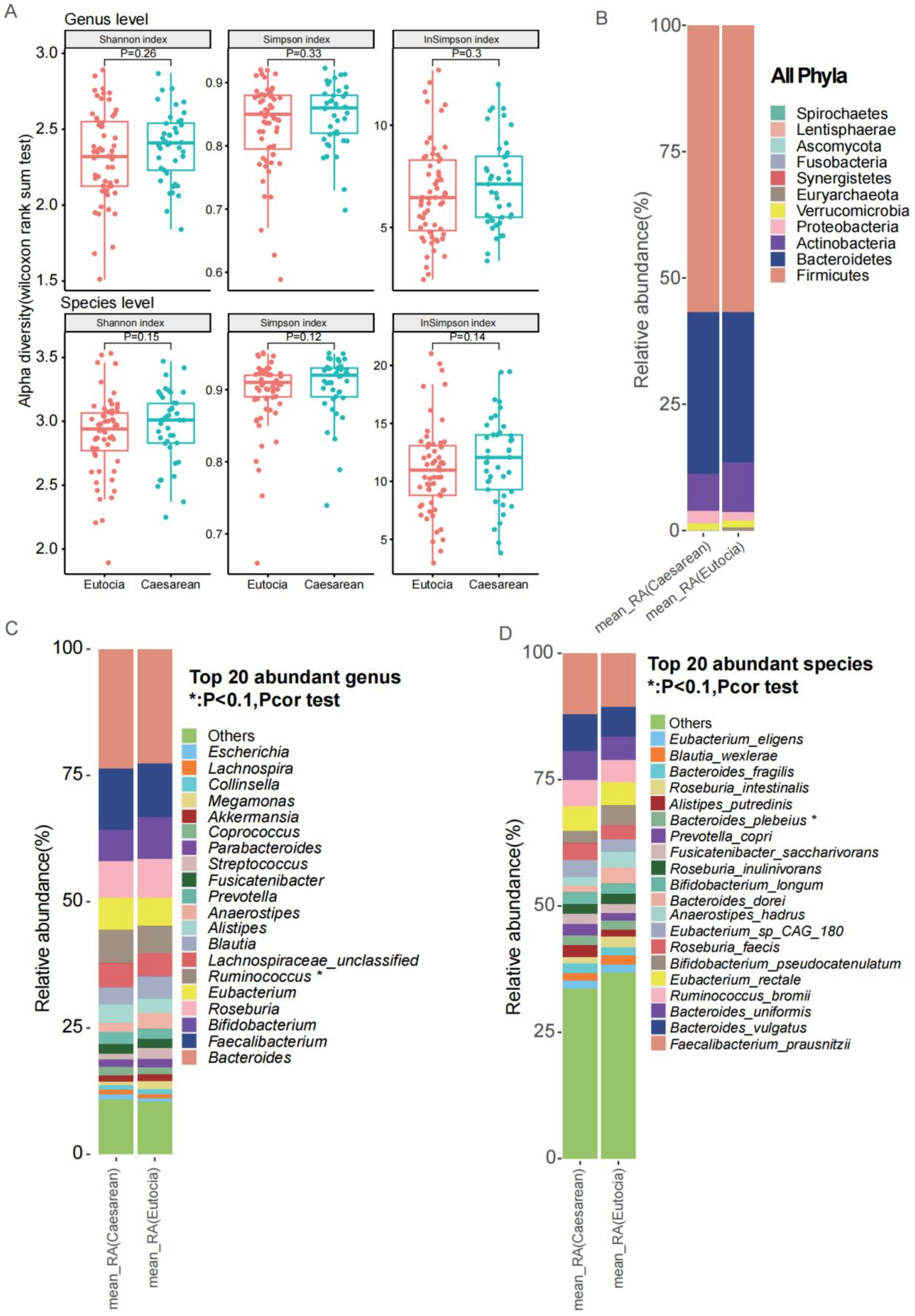

Figure S11. Diversity analysis and taxon construction under different delivery mode. (A) Diversity analysis at genus and species levels. (B) Phyla construction. (C) Top 20 abundant genera; (D) Top 20 abundant species.

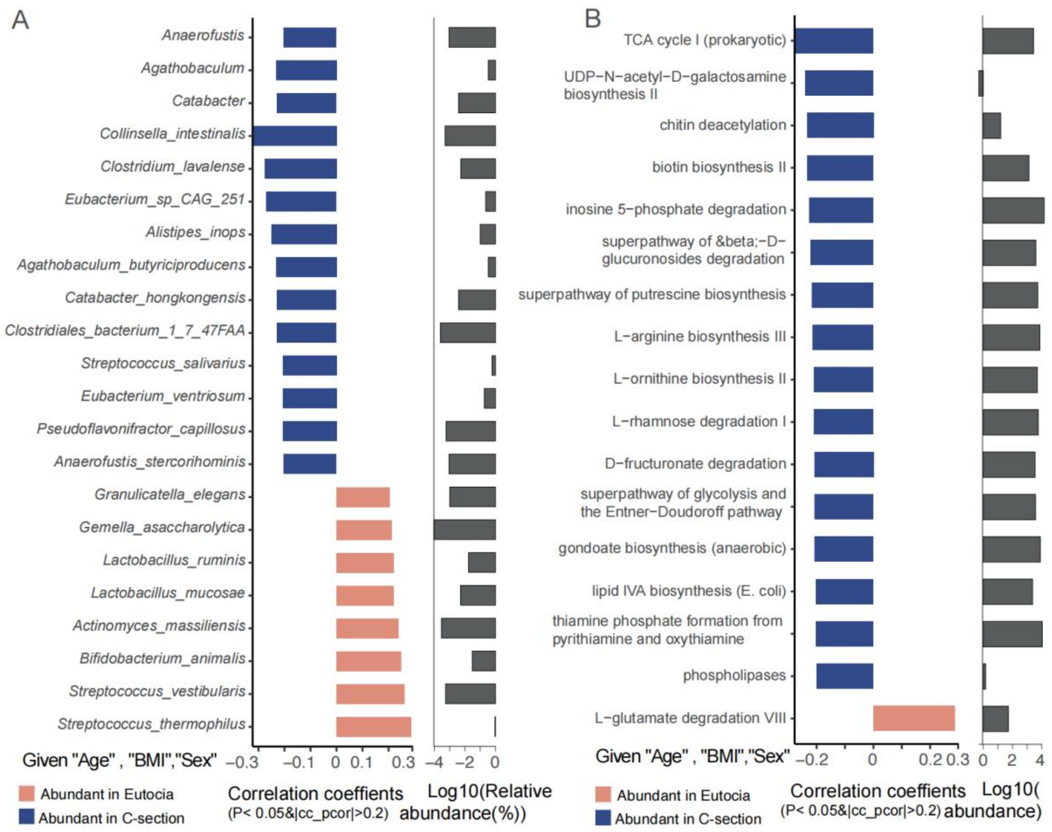

Figure S12. Significantly different species and predicted functional pathways under different delivery mode. (A) Significantly different species between Eutocia and C-section. (B) Significantly different predicted functional pathways between Eutocia and C-section. The results were given by age, BMI, and sex.

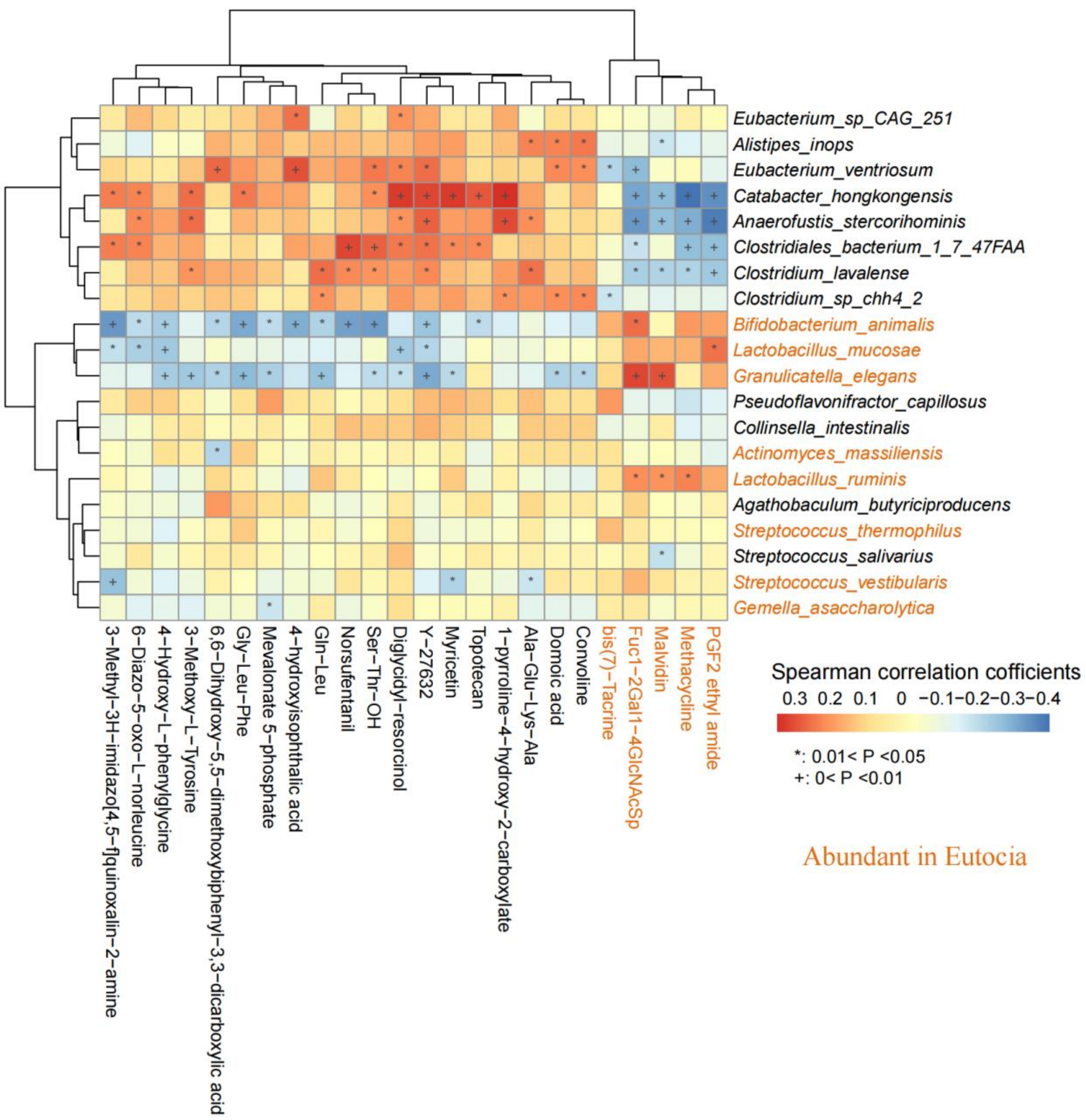

Figure S13. Correlation analysis between the significantly different species and fecal metabolites between Eutocia and C-section.

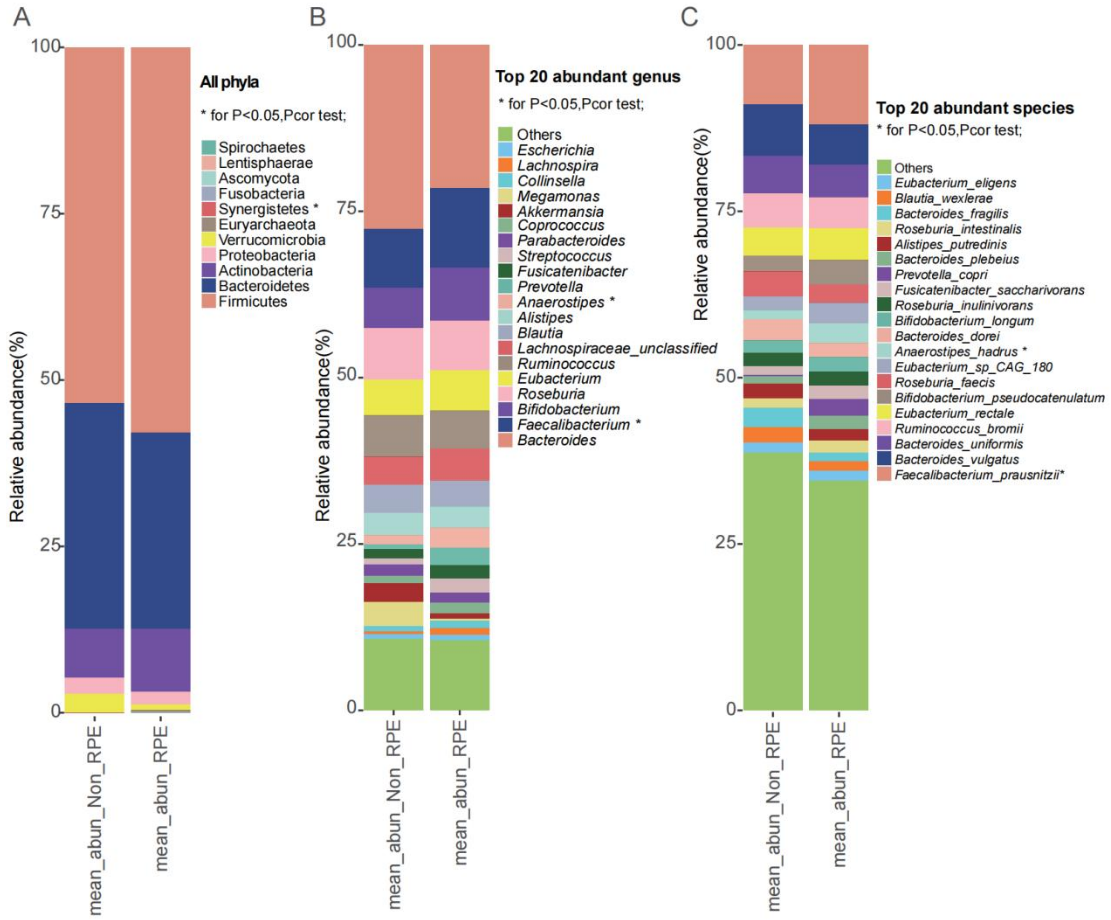

Figure S14. The structure of the taxons at different levels between children with and without regular physical exercise. (A) all phyla between two groups. (B) top 20 abundant genera between two groups. (C) top 20 abundant species between two groups.

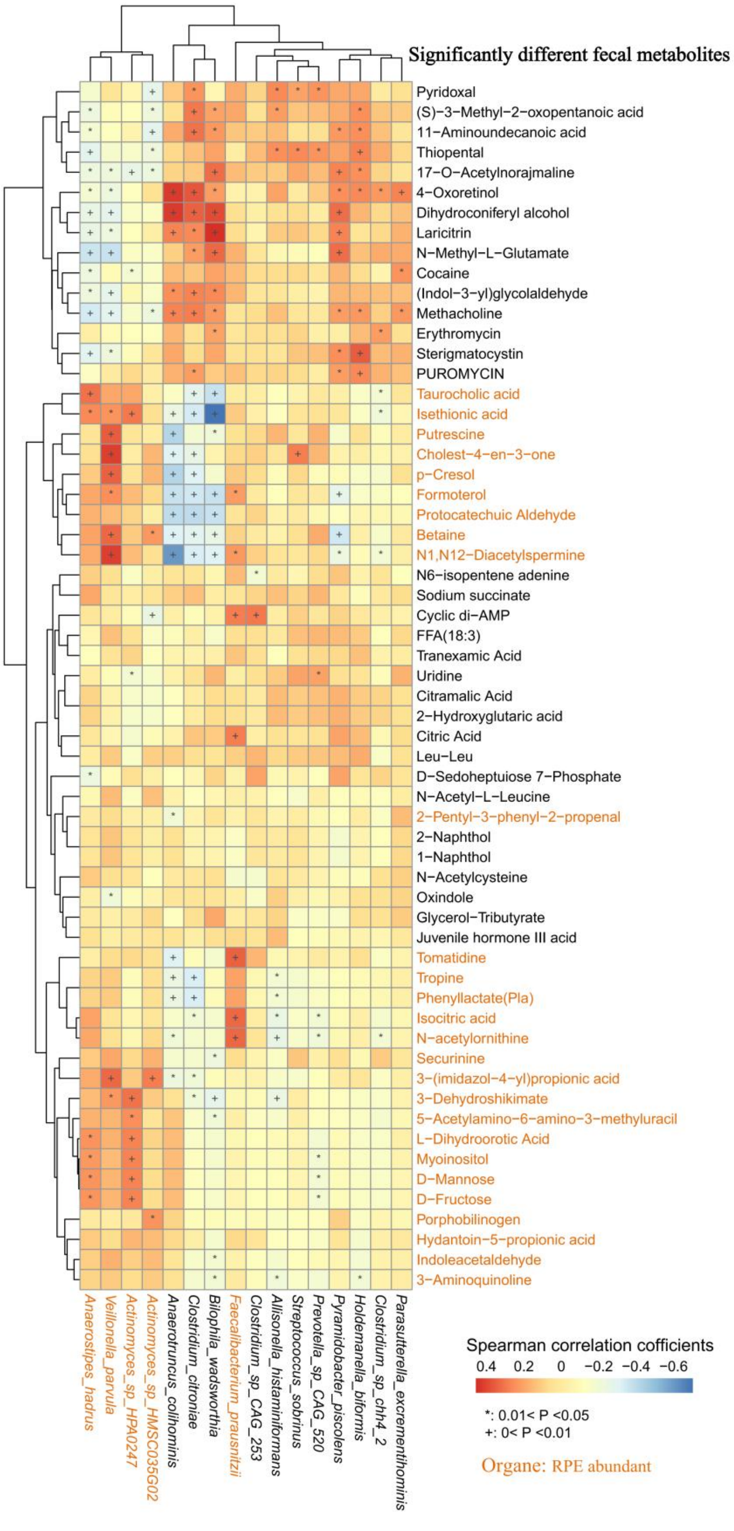

Figure S15. Correlation analysis between the significantly different species and fecal metabolites between children with and without regular exercise.

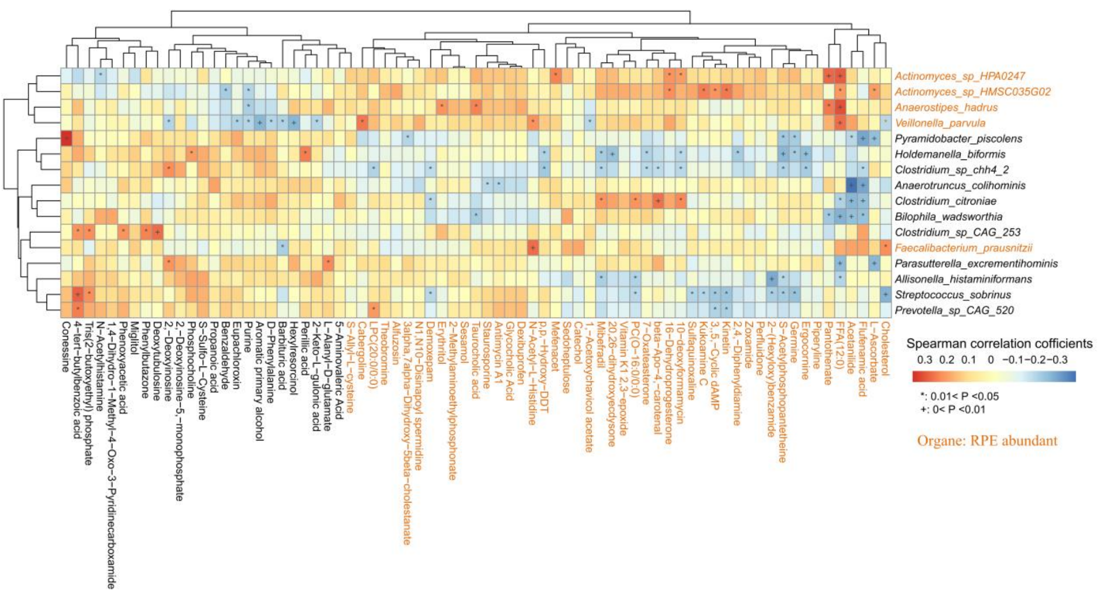

Figure S16. Correlation analysis between the significantly different species and blood metabolites between children with and without regular exercise.

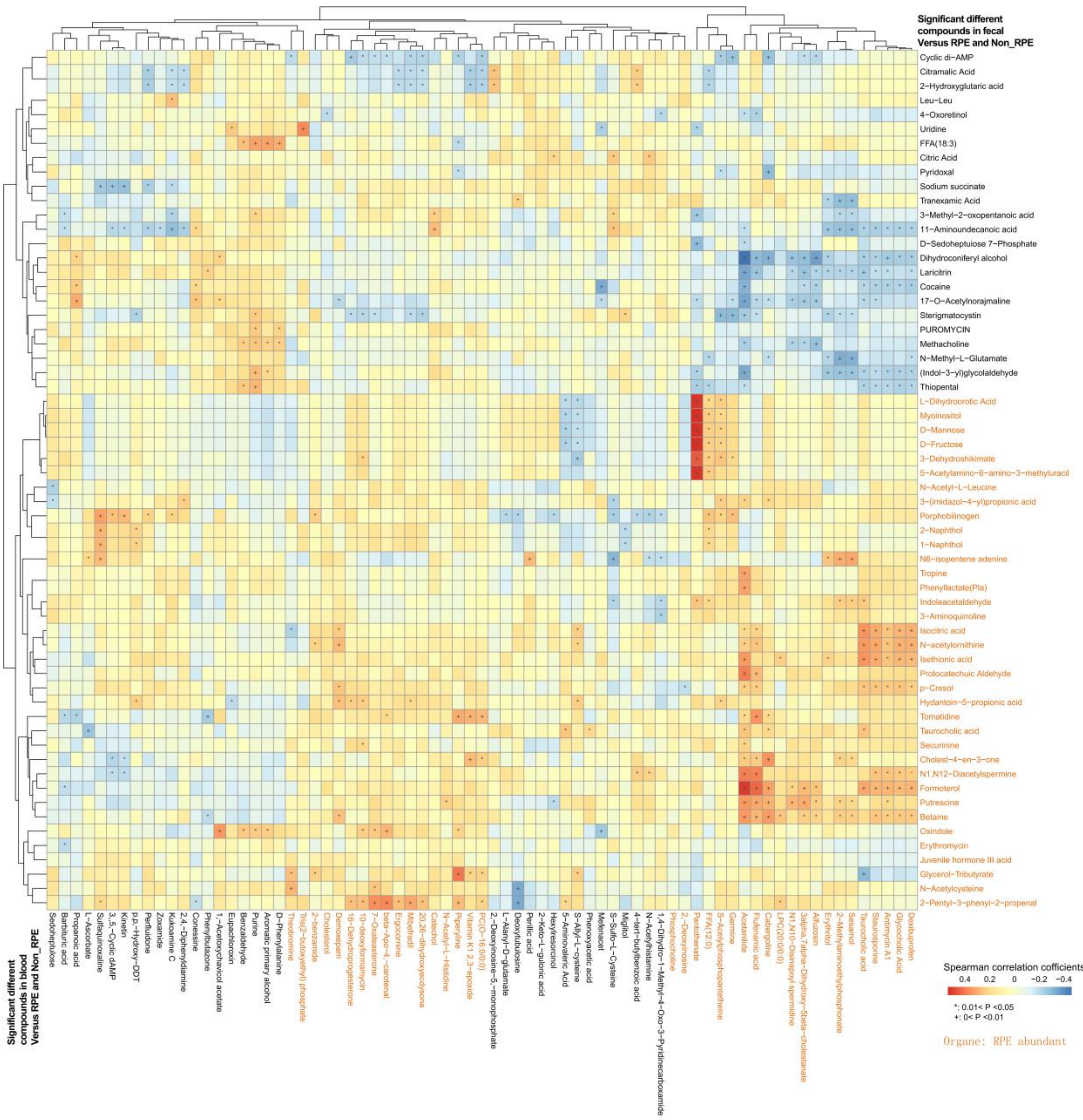

Figure S17. Correlation analysis between the significantly different fecal and blood metabolites between children with and without regular exercise.
